# ASS1 metabolically contributes to the nuclear and cytosolic p53-mediated DNA damage response

**DOI:** 10.1038/s42255-024-01060-5

**Published:** 2024-06-10

**Authors:** Lisha Qiu Jin Lim, Lital Adler, Emma Hajaj, Leandro R. Soria, Rotem Ben-Tov Perry, Naama Darzi, Ruchama Brody, Noa Furth, Michal Lichtenstein, Elizabeta Bab-Dinitz, Ziv Porat, Tevie Melman, Alexander Brandis, Sergey Malitsky, Maxim Itkin, Yael Aylon, Shifra Ben-Dor, Irit Orr, Amir Pri-Or, Rony Seger, Yoav Shaul, Eytan Ruppin, Moshe Oren, Minervo Perez, Jordan Meier, Nicola Brunetti-Pierri, Efrat Shema, Igor Ulitsky, Ayelet Erez

**Affiliations:** 1 Department of Molecular Cell Biology, Weizmann Institute of Science, Rehovot, Israel; 2 Department of Medicine D, Beilinson Hospital, Petah Tikva, Israel; 3 Telethon Institute of Genetics and Medicine, Pozzuoli, Italy; 4 Department of Immunology and Regenerative Biology, Weizmann Institute of Science, Rehovot, Israel; 5 Department of Molecular Neuroscience, Weizmann Institute of Science, Rehovot, Israel; 6 Department of Biochemistry and Molecular Biology, The Institute for Medical Research Israel-Canada, Faculty of Medicine, Hebrew University of Jerusalem, Jerusalem, Israel; 7 Department of Life Sciences Core Facilities, Weizmann Institute of Science, Rehovot, Israel; 8 The De Botton Protein Profiling Institute of the Nancy and Stephen Grand Israel National Center for Personalized Medicine, Weizmann Institute of Science, Rehovot, Israel; 9 Cancer Data Science Lab, Center for Cancer Research, National Cancer Institute, National Institute of Health, Bethesda, MD USA; 10 Department of Translational Medicine, Medical Genetics, University of Naples Federico II, Naples, Italy; 11 Scuola Superiore Meridionale (SSM, School of Advanced Studies), Genomics and Experimental Medicine Program, University of Naples Federico II, Naples, Italy

**Keywords:** Cancer metabolism, Medical genetics, Metabolism, DNA, Cell biology

## Abstract

Downregulation of the urea cycle enzyme argininosuccinate synthase (ASS1) in multiple tumors is associated with a poor prognosis partly because of the metabolic diversion of cytosolic aspartate for pyrimidine synthesis, supporting proliferation and mutagenesis owing to nucleotide imbalance. Here, we find that prolonged loss of ASS1 promotes DNA damage in colon cancer cells and fibroblasts from subjects with citrullinemia type I. Following acute induction of DNA damage with doxorubicin, ASS1 expression is elevated in the cytosol and the nucleus with at least a partial dependency on p53; ASS1 metabolically restrains cell cycle progression in the cytosol by restricting nucleotide synthesis. In the nucleus, ASS1 and ASL generate fumarate for the succination of SMARCC1, destabilizing the chromatin-remodeling complex SMARCC1–SNF5 to decrease gene transcription, specifically in a subset of the p53-regulated cell cycle genes. Thus, following DNA damage, ASS1 is part of the p53 network that pauses cell cycle progression, enabling genome maintenance and survival. Loss of ASS1 contributes to DNA damage and promotes cell cycle progression, likely contributing to cancer mutagenesis and, hence, adaptability potential.

## Abstract (web summary)

Lim, Adler et al. show that the urea cycle enzyme ASS1 can function in the nucleus to supply fumarate necessary to drive DNA damage responses.

## Main

**Extended Data Fig. 1:**
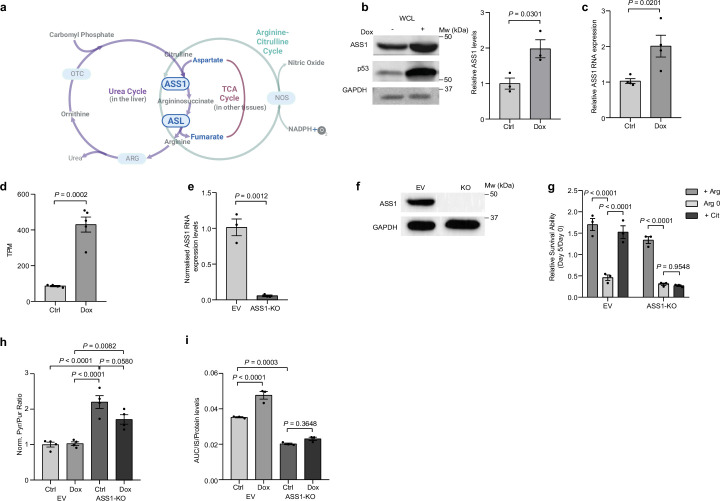
Expression of ASS1 protein and mRNA is elevated upon Dox treatment. **a**, Role of ASS1 in the urea, TCA and arginine-citrulline cycles. The scheme was created with BioRender. **b**, Protein samples from control and dox-treated HCT116 WT cells were extracted and immunoblotted for ASS1 and p53. Loading control - GAPDH. (*n* = 3 independent experimetns) **Right panel:** Quantitative analysis of ASS1 protein expression levels, normalised to GAPDH. **c**, *ASS1* RNA expression levels of Ctrl or Dox-treated NF were quantified using RT-PCR. (*n* = 4 independent experiments) **d**, RNA seq analysis of *ASS1* levels in control and dox-treated HCT116 cells (upper panel), and normal fibroblasts (lower panel) (*n* = 5 biologically independent samples) **e**, HCT116 ASS1-KO cells were generated using CRISPR-Cas9 and validated for *ASS1* RNA expression using qRTPCR. (*n* = 3 independent experiments) **f**, HCT116 ASS1-KO cells were generated using CRISPR-Cas9 and validated for ASS1 protein expression using immunoblotting. (*n* = 3 independent experiments). **g**, HCT116 ASS1-KO cells were generated using CRISPR-Cas9 and validated for ASS1 enzymatic activity using a proliferative assay, in which citrulline supplementation (200 mg/ml), in the absence of arginine, does not rescue survival of cells deficient in ASS1. (*n* = 3 independent experiments) (P-values: EV Arg0-+Arg:<0.0001; EV Arg0-+Cit:<0.0001; ASS1-KO Arg0-+Arg:<0.0001; ASS1-KO Arg0-+Cit: 0.9548) **h**, Nucleotide levels of Ctrl or Dox-treated HCT116 ASS1-EV (EV) and ASS1-KO cells were measured and quantified as a normalised Pyr/Pur ratio. (*n* = 4 independent experiments) (P-values: EV Ctrl-ASS1-KO Ctrl:<0.0001; EV Dox-ASS1-KO Ctrl:<0.0001); EV Dox-ASS1-KO Dox:0.0082; ASS1-KO Ctrl-ASS1-KO Dox:<0.0001) **i**, Fumarate levels of Ctrl or Dox-treated HCT116 ASS1-EV (EV) and ASS1-KO cells were measured and quantified by GC/MS. (*n* = 3 biologically independent samples). (P-value: EV Ctrl-EV Dox:<0.0001; EV Ctrl-ASS1-KO Ctrl:0.0003; ASS1-KO Ctrl-ASS1-KO Dox:0.3648). Data are represented as the mean ± s.e.m. ns, not significant. *P* values were determined by two-way ANOVA with Tukey’s multiple-comparison test (**g**), one-way ANOVA with Tukey’s multiple-comparison test (**h** and **i**) or unpaired two-tailed Student’s *t*-test (**b**, **c**, **d** and **e**).

The urea cycle is the primary metabolic pathway in mammals for detoxifying excess nitrogen by converting ammonia to excreted urea. The urea cycle enzyme ASS1 conjugates nitrogen from aspartate and citrulline to form argininosuccinate. Subsequently, argininosuccinate is cleaved by argininosuccinate lyase (ASL) to generate fumarate, which leaves the urea cycle to enter the tricarboxylic acid cycle, and arginine, which continues in the cycle and is cleaved by arginase to form ornithine and urea^1^. Outside the liver, ASS1 participates with ASL in the cytosolic arginine–citrulline cycle to supply the cellular needs for arginine and its downstream metabolites^1^ and fumarate (Extended Data Fig. 1a).

Individuals with germline pathogenic variants in the gene encoding ASS1 have citrullinemia type I (CTLN-I). These patients may present with hyperammonemia, clinically manifesting in seizures, lethargy, coma and potentially death^2^. In contrast to the established importance of ASS1 functionality to human health, silencing ASS1 expression by promoter methylation has been observed in many cancer types^3^. We have shown that decreased activity of ASS1 promotes cancerous proliferation by increasing aspartate availability for pyrimidine nucleotide synthesis^4^. Furthermore, we found that high pyrimidine levels following the downregulation of ASS1 generate a nucleotide imbalance that can increase tumor immunogenicity and responsiveness to immunotherapy^5,6^. Thus, ASS1 regulation of pyrimidine levels has diagnostic and therapeutic implications for cancer patients.

Interestingly, it has been shown recently in colon cancer cells (HCT116) that the promoter region of human *ASS1* contains a binding site for p53 (ref. ^7^). p53 directly promoted the expression of *ASS1* mRNA and protein under genotoxic stress induced by X-ray irradiation or doxorubicin (Dox)^7^. Given the importance of ASS1 in maintaining nucleotide pools and the centrality of p53 in regulating the response to DNA damage, we reasoned that the p53-dependent regulation of ASS1 may contribute to the cellular response to DNA damage.

## Results

### Loss of cytosolic ASS1 causes DNA damage

To dissect the potential role of ASS1 in the p53-regulated DNA damage response, we induced DNA damage in HCT116 cells carrying wild-type (WT) p53 using Dox^8^. Following treatment with Dox, and consistent with previous findings^7^, western blot analyses showed a significant increase in ASS1 expression that coincides with p53 protein upregulation (Extended Data Fig. 1b). RNA analysis further confirmed that the upregulation in *ASS1* expression following Dox is in colon cancer cells as well as in normal fibroblasts (Extended Data Fig. 1c,d). To determine the specific role of ASS1 in the DNA damage response, we knocked out *ASS1* in HCT116 cells using the CRISPR–Cas9 system^9^ and verified ASS1 knockout (KO) at the RNA and protein levels (Extended Data Fig. 1e,f). We evaluated the KO functionally with a metabolic assay of ASS1; given that ASS1 is required to synthesize arginine from citrulline, under arginine depletion, cells without ASS1 cannot proliferate even with citrulline supplementation^10^. Indeed, ASS1-KO cells could not survive in an arginine-depleted medium, and citrulline supplementation rescued only the cells expressing ASS1, providing functional validation of the absence of ASS1 in the ASS1-KO cells (Extended Data Fig. 1g).

**Fig. 1:**
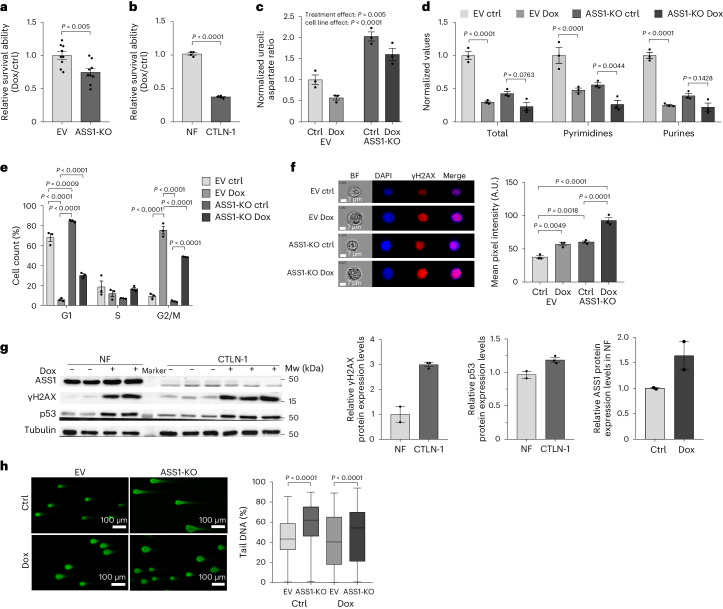
ASS1 is essential for the p53 regulation of the cell cycle and genome integrity. **a**, Quantification of the relative cell survival ability of HCT116 EV and ASS1-KO cells with and without Dox treatment. *n* = 8 independent experiments; EV, empty vector; ctrl, control. **b**, Quantification of the relative cell survival ability of normal fibroblasts (NF) and ASS1-deficient patient-derived skin fibroblasts (CTLN-I) with and without Dox. *n* = 4 independent experiments. **c**, Uracil:aspartate ratio of HCT116 EV and ASS1-KO cells with and without Dox. Treatment effect was significant (*F*_1,8_ = 14.7, *P* = 0.005) and similar in both cell lines (non-significant interaction between cell line and treatment; *F*_1,8_ < 0.01, *P* = 0.984). *n* = 3 biologically independent samples. **d**, Normalized total, pyrimidine and purine nucleotide levels as measured by LC–MS for HCT116 EV and ASS1-KO cells, with and without Dox (total: EV ctrl-EV Dox, *P* < 0.0001; ASS1-KO ctrl-ASS1-KO Dox, *P* = 0.0763. Pyrimidines: EV ctrl-EV Dox, *P* < 0.0001; ASS1-KO ctrl-ASS1-KO Dox, *P* = 0.0044. Purines: EV ctrl-EV Dox, *P* < 0.0001; ASS1-KO ctrl-ASS1-KO Dox, *P* = 0.1428). *n* = 3 biologically independent samples. **e**, Cell cycle of HCT116 EV and ASS1-KO cells, with and without Dox (G1: EV ctrl-EV Dox, *P* < 0.0001; EV ctrl-ASS1-KO ctrl, *P* *=* 0.0009; EV Dox-ASS1-KO ctrl, *P* < 0.0001; EV Dox-ASS1-KO Dox, *P* < 0.0001. G2M: EV ctrl-EV Dox, *P* < 0.0001; EV Dox-ASS1-KO ctrl, *P* < 0.0001; EV Dox-ASS1-KO Dox, *P* < 0.0001; ASS1-KO ctrl-ASS1-KO Dox, *P* < 0.0001). *n* = 3 biologically independent samples. **f**, Representative immunofluorescence images of γH2AX levels (Alexa Fluor 647) in control and Dox-treated HCT116 EV and ASS1-KO cells. Right panel: mean pixel intensity quantification (EV ctrl-EV Dox, *P* = 0.0049; EV ctrl-ASS1-KO ctrl, 0.0018; EV ctrl-ASS1-KO Dox, *P* < 0.0001; ASS1-KO ctrl-ASS1-KO Dox, *P* < 0.0001). *n* = 3 biologically independent samples. **g**, NF and ASS1-deficient patient-derived skin fibroblasts (CTLN-I) treated with and without Dox were immunoblotted for γH2AX, p53 and ASS1. Tubulin: loading control; MW, molecular weight. Right panel: Quantification of γH2AX and p53 protein expression levels comparing untreated NF and untreated CTLN-I cells; and for ASS1 protein expression levels in treated and untreated NF (NF, *n* = 2; CTLN-I, *n* = 3 biologically independent samples). **h**, Tail DNA percentage (%), in HCT116 EV and ASS1-KO cells with and without Dox treatment visualized using the comet assay. Representative fluorescent images for each sample are shown. Right panel: tail DNA percentage quantification. *n* = 600 biologically independent cells across 3 biologically independent samples (ctrl EV-ASS1-KO, *P* < 0.0001; Dox EV-ASS1-KO, *P* < 0.0001). Data are represented as the mean ± s.e.m. *P* values were determined by two-way ANOVA with Tukey’s honest significant differences method (in **c** and **e**), two-way ANOVA with Tukey’s multiple-comparison test (in **d**), ordinary one-way ANOVA with Sidak’s multiple-comparison test (in **f** and **h**) or unpaired two-tailed Student’s *t*-test (in **a**, **b** and **g**). Boxplot for **h** is min–max with the line at the median; shaded area, 50^th^ percentile of each dataset. Source data

To investigate whether ASS1 provides a survival advantage following DNA damage, we measured survival after Dox treatment in colon cancer cells with and without ASS1. ASS1-deficient colon cancer cells had significantly lower survival following DNA damage than parental cells expressing ASS1 (Fig. 1a). Given that cancer cells can hijack a physiological survival mechanism and exploit it for their benefits^11^, we measured cell survival following Dox application in non-transformed control and ASS1-deficient skin fibroblasts biopsied from healthy patients and those with CTLN-1, respectively. Similar to our results in cancer cells, ASS1-deficient skin fibroblasts had significantly lower survival following Dox treatment than control fibroblasts (Fig. 1b).

Previously, we demonstrated that ASS1 expression restricts aspartate availability for the carbamoyl-phosphate synthetase 2, aspartate transcarbamylase and dihydroorotase enzymatic complex (CAD) to synthesize pyrimidine nucleotides for the generation of DNA and RNA that support proliferation^4,6^. Thus, we reasoned that ASS1 upregulation by p53 may decrease aspartate levels following DNA damage, hampering nucleotide synthesis and metabolically pausing cell cycle progression. To test this hypothesis, we analyzed the levels of uracil relative to aspartate in colon cancer cells with and without ASS1, following Dox treatment. We found that DNA damage significantly reduced the levels of uracil to aspartate in colon cancer cells expressing ASS1. Conversely, in cells lacking ASS1, the levels of uracil to aspartate were higher and did not change substantially upon DNA damage (Fig. 1c). Following Dox treatment or ASS1 loss, we found a significant reduction in total nucleotide levels in colon cancer cells (Fig. 1d). Specifically, in ASS1-KO cells, we found, as expected^4–6^, that the increase in aspartate availability led to an increase in the pyrimidine-to-purine ratio (Extended Data Fig. 1h). In addition, although the total nucleotide levels were not significantly altered in ASS1-KO cells following treatment with Dox, pyrimidine synthesis decreased significantly more than purine synthesis, decreasing the pyrimidine-to-purine ratio (Fig. 1d and Extended Data Fig. 1h).

p53 regulates cell cycle progression following DNA damage by Dox by lengthening the G2/M phases of the cell cycle^12^. Following DNA damage, we reasoned that the p53-dependent increase of ASS1 expression might metabolically regulate cell cycle progression by controlling nucleotide levels. Therefore, we evaluated the cell cycle distribution of Dox-treated colon cancer cells. Following Dox treatment, in cells with and without ASS1, the G1 phase was shortened and the G2/M phase was protracted compared to untreated cells (Fig. 1e). Notably, in untreated colon cancer cells without ASS1, the G1 phase was prolonged significantly more than in cells expressing ASS1 and the G2 phase was shorter (Fig. 1e). Following Dox treatment, the percentage of cells at G1 phase was higher and that of cells at G2 was lower in the ASS1-KO cells (Fig. 1e). These results suggest that following DNA damage, ASS1 expression is upregulated as part of the p53-DNA damage response (DDR) to generate a nucleotide depletion that can pause the progression of the cell cycle. These p53-related effects are less prominent in colon cancer cells with ASS1 loss. The decreased nucleotide synthesis and prolonged G1 phase in cancer cells with ASS1 deficiency, even without induction of DNA damage, suggests that ASS1 loss may delay entrance to the cell cycle by dysregulating nucleotide synthesis even at a basal state (Fig. 1d,e).

γH2AX has been identified as a specific and sensitive marker for DNA damage and DDR^13^. Indeed, we found that γH2AX foci are significantly increased in the nuclei of the Dox-treated control cancer cells compared to untreated cells (Fig. 1f). Notably, independent of Dox treatment, γH2AX levels were higher in ASS1-KO cells than in cells expressing ASS1 (Fig. 1f). Similarly, in skin fibroblasts from patients that are deficient in ASS1 (CTLN-I), baseline γH2AX levels were higher than in normal fibroblasts (Fig. 1g). Furthermore, at basal state, p53 levels were also significantly higher in CTLN-I fibroblasts, suggesting that ASS1 loss by itself may induce DNA damage (Fig. 1g). To test whether the loss of ASS1 directly causes DNA damage, we performed a comet assay on colon cancer cells with and without ASS1 (ref. ^14^). Consistent with the γH2AX foci levels (Fig. 1f), we observed longer comet tails representing more DNA damage in untreated and Dox-treated ASS1-KO colon cancer cells than in control cells (Fig. 1h).

These data suggest that ASS1 loss contributes to DNA damage at a steady state, and more so following Dox treatment.

### ASS1 presence in the nucleus increases following DNA damage

**Fig. 2:**
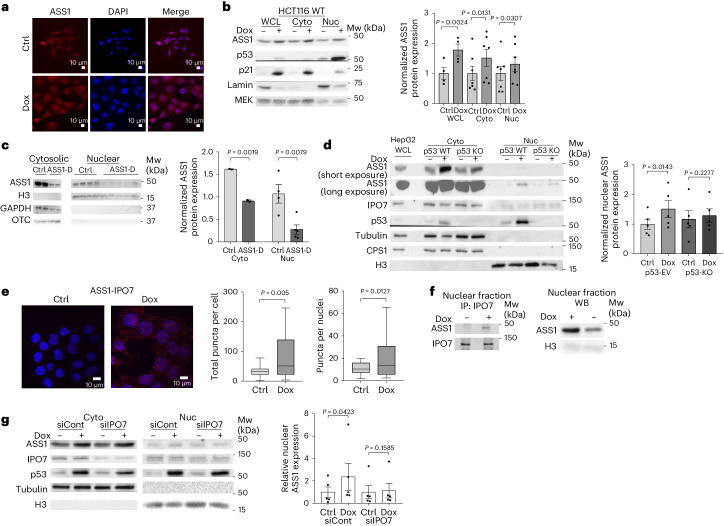
ASS1 generates fumarate in the nucleus. **a**, Control and Dox-treated HCT116 WT cells were stained for ASS1 with Alexa Fluor 594 (red). *n* = 3 biologically independent experiments. **b**, Cytosolic and nuclear fractions of control and Dox-treated HCT116 WT cells were immunoblotted. Markers: lamin, nuclear; MEK, cytoplasmic Right panel: quantification of nuclear ASS1 normalized to H3 (Nuc), cytosolic ASS1 normalized to GAPDH (Cyto) and WCL ASS1 normalized to GAPDH (WCL). *n* = 4 biologically independent experiments. **c**, Livers from control (ASS^Flox/Flox^) and Alb-cre ASS1 mice (ASS1-D) were fractionated and immunoblotted for ASS1. Markers: H3, nuclear; GAPDH, cytoplasmic; OTC, mitochondrial. The faint band in cytosolic ASS1-D probably results from other liver cells expressing ASS1. *n* = 4 biologically independent samples. Right panel: quantification of nuclear ASS1 levels relative to H3 (Nuc) and cytosolic ASS1 levels normalized to GAPDH (Cyto). **d**, Control and Dox-treated HCT116 p53 WT and p53-KO cells were fractionated into cytosol (Cyto) and nuclear (Nuc) fractions and immunoblotted. Markers: H3, nuclear; tubulin, cytoplasmic; CPS1, mitochondrial. *n* = 5 biologically independent samples. Right panel: quantification of nuclear ASS1 levels relative to H3. **e**, ASS1–IPO7 interactions in control and Dox-treated HCT116 ASS1-EV and ASS1-KO cells. Right panel: quantification of total and nuclear puncta. *n* = 300 biologically independent cells across three biologically independent samples. **f**, Immunoprecipitation of IPO7 was performed on the nuclear fraction of control and Dox-treated HCT116 WT cells and immunoblotted (left). The nuclear lysate was immunoblotted for ASS1 and H3 (nuclear marker) (right). *n* = 3 independent experiments. **g**, Cytosolic and nuclear fractions of control and Dox-treated HCT116 WT cells transfected with scrambled or IPO7-targeted siRNA were immunoblotted for ASS1, p53 and IPO7. Markers: H3, nuclear; tubulin, cytoplasmic. Right panel: quantification of nuclear ASS1 protein expression levels, normalized to H3, and relative to ctrl. *n* = 5 independent experiments. Data are represented as the mean ± s.e.m. *P* values were determined by paired two-tailed Student’s *t*-test (in **b** and **d**), unpaired two-tailed Student’s *t*-test (in **c** and **e**) and two-tailed ratio paired *t*-test (in **g**). Boxplot in **e** represents min–max, with the line at the median and the shaded area representing the 50^th^ percentile of each dataset. Source data

**Extended Data Fig. 2:**
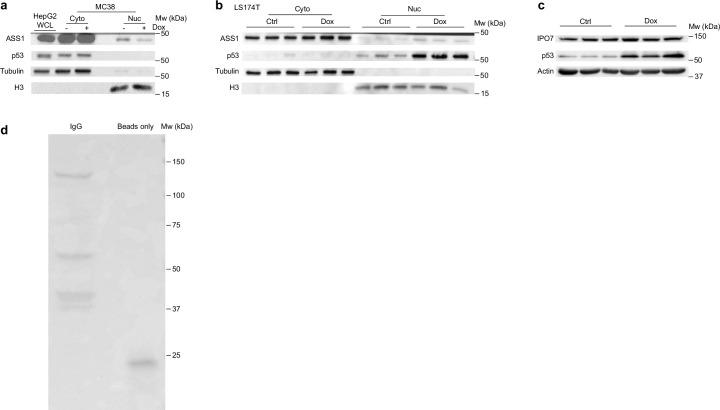
Nuclear expression of ASS1 is dependent on WT. **a**, MC38 cells, colon cancer cells with mutated p53, were incubated with (+) or without Dox (-) for 2 hrs, and after 48 hrs, fractionated into nuclear (Nuc) and cytoplasmic (Cyto) fractions and immunoblotted for ASS1 and p53. Tubulin and H3 were used as a cytoplasmic or nuclear marker, respectively. (*n* = 3 biologically independent samples). HepG2 WCL was used as a positive control for CPS1 **b**, LS-174-T cells, colon cancer cells with wild-type p53, were incubated with (Dox) or without Dox (Ctrl) for 2 hrs, and after 48 hrs, fractionated into nuclear (Nuc) and cytoplasmic (Cyto) fractions and immunoblotted for ASS1 and p53. Tubulin and H3 were used as a cytoplasmic or nuclear marker, respectively. (*n* = 3 biologically independent samples). **c**, HCT116 WT cells were incubated with (Dox) or without Dox (Ctrl) for 2 hrs, and after 48 hrs, protein was extracted and immunoblotted for ASS1, IPO7 and p53. β-actin was used as a loading control. (*n* = 3 biologically independent samples) **d**, HCT116 WT cells were incubated with (+) or without Dox (-) for 2 hrs, and fractionated after 48 hrs. IgG and beads only controls were also immunoblotted for IPO7. (*n* = 3 independent experiments).

To further characterize the cellular effects of ASS1, we performed immunofluorescence staining for ASS1 following Dox treatment. As expected, Dox increased nuclear size in all surviving cells treated, reflecting the induction of DNA damage^15^. However, although ASS1 is an established cytosolic enzyme not known to have a nuclear localization signal^16^, we unexpectedly observed nuclear expression of ASS1 in untreated HCT116 colon cancer cells, with a robust increase following Dox treatment (Fig. 2a). By fractionating HCT116 cells into cytosolic and nuclear compartments, we confirmed that both ASS1 and p53 accumulated in the nuclei of HCT116 colon cancer cells after Dox treatment (Fig. 2b). To interrogate whether ASS1 nuclear localization might be cancer-dependent, we fractionated hepatocytes from livers of WT (ASS^Flox/Flox^) and liver-specific ASS1-KO (Alb-cre) mice^5^. We confirmed that ASS1 is also present in the nucleus of normal hepatocytes (Fig. 2c). To determine whether the elevation in nuclear ASS1 might be p53-dependent, we used HCT116 cells with WT levels of p53 and an isogenic line in which p53 expression was disrupted^17^. We found that p53 expression significantly elevates ASS1 levels in the nucleus following Dox-induced DNA damage (Fig. 2d). By contrast, in mouse colon cancer cells (MC38) with mutated p53 (ref. ^18^), we found no elevation in ASS1 nuclear localization following Dox treatment (Extended Data Fig. 2a), whereas in LS-174-T colon cancer cells expressing WT p53 (ref. ^19^), we again found nuclear expression of ASS1 following treatment with Dox **(**Extended Data Fig. 2b).

To understand how ASS1 enters the nucleus, we found in the literature that importin 7 (IPO7) can facilitate the import of proteins that lack a nuclear localization signal, including p53-modulated proteins^20–22^. We first tested whether IPO7 is regulated by p53 following DNA damage and found that Dox-induced DNA damage did not change the total levels of IPO7 (Extended Data Fig. 2c). Moreover, there were no significant differences in the cytosolic and nuclear levels of IPO7 in cancer cells with loss of p53 with and without Dox treatment compared to cancer cells expressing WT p53 (Fig. 2d). Therefore, we next evaluated the effect that DNA damage has on the interaction between IPO7 and ASS1. Mass spectrometry (MS) analysis of nuclear proteins pulled down by ASS1 following Dox treatment suggested an interaction between IPO7 and ASS1 (Supplementary Table 2). Using immunoprecipitation and a proximity ligation assay (PLA), we further confirmed that ASS1 interacts with IPO7 and that this interaction increases upon Dox treatment (Fig. 2e,f and Extended Data Fig. 2d). Furthermore, we found that in HCT116 colon cancer cells treated with siIPO7, nuclear ASS1 levels did not significantly change following DNA damage compared to cancer cells treated with siControl (Fig. 2g). Thus, our results suggest that DNA damage augments the interaction between ASS1 and IPO7, facilitating the presence of ASS1 in the nucleus.

### ASS1 and ASL presence in the nucleus generate fumarate

**Fig. 3:**
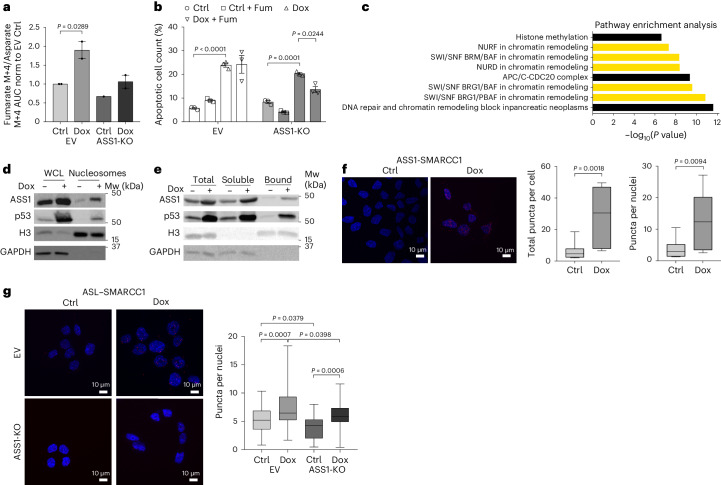
Nuclear ASS1 contributes to fumarate generation and interacts with SMARCC1. **a**, Isotopic tracing of labeled M + 4 fumarate (AUC) generated from M + 4 aspartate (AUC) in the nuclei was performed on control and Dox-treated HCT116 ASS1-EV (EV) and ASS1-KO cells. *n* = 2 biologically independent samples, ASS1-KO, *n* = 1. **b**, Cell survival of control and Dox-treated HCT116 ASS1-EV (EV) and ASS1-KO cells were incubated with (+Fum) or without Fumarate (1 mM) using Apotracker. *n* = 3 biologically independent samples (EV ctrl-Dox, *P* < 0.0001; ASS1-KO ctrl-Dox, *P* < 0.0001; ASS1-KO Dox-Dox+Fum, *P* = 0.0244). **c**, Pathway analysis of ASS1 protein interactors from the nuclear fraction of HCT116 WT cells, after immunoprecipitation–MS using ASS1 antibody of the nuclear fraction of HCT116 WT cells. Chromatin-remodeling pathways related to SWI/SNF are highlighted in yellow. *n* = 3 biologically independent samples. **d**, Nucleosomes from control and Dox-treated HCT116 WT cells isolated by MNase digestion and immunoblotted for ASS1 and p53. Markers: H3, nuclear; GADPH, cytoplasmic. *n* = 3 independent experiments. **e**, Control and Dox-treated HCT116 WT cells assayed for the total, soluble and chromatin-bound fraction and immunoblotted for ASS1 and p53. Markers: H3, nuclear; GADPH, cytoplasmic. *n* = 3 independent experiments. **f**, Interactions between ASS1 and SMARCC1 of control and Dox-treated HCT116 WT cells visualized using PLA. Right panel: quantification of total puncta and nuclear puncta. *n* = 100 biologically independent cells across three biologically independent samples. **g**, interactions between ASL and SMARCC1 of control and Dox-treated HCT116 ASS1-EV (EV) and ASS1-KO cells visualized using a PLA. Right panel: quantification of nuclear puncta. *n* = 200 biologically independent cells across three biologically independent samples. Data are represented as the mean ± s.e.m. ns, not significant. *P* values were determined by one-way ANOVA with Tukey’s multiple-comparison test (in **g**), two-way ANOVA on the log_10_ transformed values (in **b**) or unpaired two-tailed Student’s *t*-test (in **a**, **c** and **f**). Boxplot in **f** and **g** is min–max, with the line at the median and the shaded area representing the 50^th^ percentile of each dataset. Source data

**Extended Data Fig. 3:**
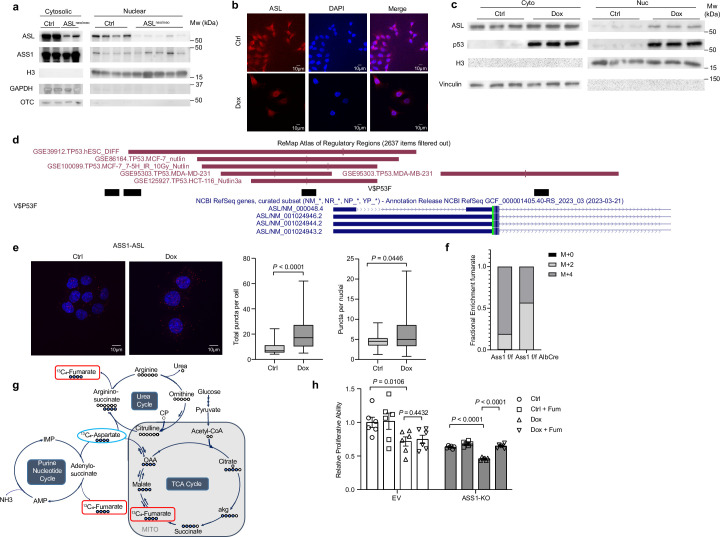
ASL participates in the nuclear generation of fumarate. **a**, Livers from control and ASL-KO (ASL^neo/neo^) were fractionated and immunoblotted for ASS1. H3 and GAPDH are used as a nuclear and cytoplasmic marker, respectively. (*n* = 4 biologically independent samples) **b**, Control and dox-treated HCT116 WT cells were stained for ASL using Alexa Fluor 594 (red). Nuclei were counter-stained with DAPI (blue). Scale bar, 10 μm. (*n* = 3 independent experiments) **c**, Control and dox-treated HCT116 WT cells were fractionated and immunoblotted for ASL and p53. Markers: Vinculin - cytoplasmic; H3 nuclear (*n* = 3 biologically independent samples) **d**, 1,100 bp surrounding the ASL transcription start site (GRCh38 chr7:66,075,357-66,076,456) taken from the UCSC genome browser (http://genome.ucsc.edu)^6^ The tracks shown: ReMap Altas of Regulatory regions filtered for TP53, TP63, TP73 binding sites (only TP53 are found)^62^; MatInspector (Genomatix Genome Analyzer^63^)predicted P53 binding sites; and RefSeq mRNAs. A binding site in HCT116 can be found in the proximal promoter, which overlaps a predicted binding site as well as binding peaks found in other cell lines. **e**, **Left panel:** Interactions between ASS1 and ASL were visualised in control and dox-treated HCT116 ASS1-EV (EV) and ASS1-KO cells using a proximity ligation assay. Scale bar, 10 μm. (P-values: Total: <0.0001, Nuclear: 0.0446) **Right panel**: Quantification of total puncta and nuclear puncta. (*n* = 100 biologically independent cells). **f**, Fractional labelling of fumarate M + 4/all masses generated from ^13^C_4_-aspartate in purified nuclei isolated from mice’s livers with and without ASS1. (Ass1^Flox/Flox^ ; Ass1^Flox/Flox^ AlbCre: *n* = 1 in each group. biologically independent mouse) **g**, Schematic depiction of the potential sources of fumarate M + 4 from labelled ^13^C-aspartate M + 4. **h**, Control and dox-treated HCT116 ASS1-EV (EV) and ASS1-KO cells were incubated with (+ Fum) or without Fumarate (1 mM), and survival was measured and quantified using XTT. (*n* = 6 biologically independent experiments) (P-value: EV Ctrl-EV Dox:0.0195; EV Dox-EV Dox+Fum:0.4432; ASS1-KO Ctrl-ASS1-Dox:<0.0001; ASS1-KO Dox-ASS1-KO Dox+Fum:<0.0001) Data are represented as the mean ± s.e.m. ns, not significant. *P* values were determined by RM one-way ANOVA (**h**), unpaired two-tailed Student’s *t*-test (**e**), or Tukey’s multiple-comparison test (**f**). Box plot for **e** is min-max with the line at the median and the shaded area represents 50 percentile of each dataset.

Given that the prominent metabolic roles of ASS1 for generating arginine and fumarate require the expression of ASL (Extended Data Fig. 1a), we next explored the expression of ASL in the nucleus. Interestingly, we demonstrated that ASL is present in the nuclei of hepatocytes from livers of WT mice but not from ASL-deficient controls^1,23^ (Extended Data Fig. 3a). Like ASS1, the nuclear and cytosolic levels of ASL in colon cancer cells increased upon Dox-induced DNA damage (Extended Data Fig. 3b,c). To evaluate whether ASL elevation following Dox treatment depends on p53 activity, we analyzed the sequence of the *ASL* promoter. We found putative binding sites for p53 and chromatin immunoprecipitation with sequencing peaks that localized to the *ASL* promoter (Extended Data Fig. 3d). Furthermore, we demonstrate that the proximity between ASL and ASS1 increases following Dox treatment in the cytosol and nucleus (Extended Data Fig. 3e). To confirm that the nuclear localization of ASS1 indeed contributes to fumarate synthesis, we incubated nuclei isolated from livers of mice with and without ASS1 expression with ^13^C_4_-aspartate and found decreased levels of M + 4 fumarate in nuclei without ASS1 (Extended Data Fig. 3f,g and Supplementary Table 1). To evaluate the increased requirement for fumarate following DNA damage, we incubated nuclei isolated from cancer cells with and without ASS1 with isotopically labeled ^13^C_4_-aspartate. We found that following Dox exposure, nuclei with ASS1 generated higher M + 4 labeled fumarate to Aspartate levels (Fig. 3a). Notably, cells with ASS1-KO still generated fumarate, probably from malate as has been previously described^24^.

Interestingly, elevated levels of fumarase and fumarate were found in the nucleus to associate with DNA damage repair^24^. Therefore, we reasoned that the nuclear role of ASS1–ASL may enable survival following DNA damage by generating fumarate. To test this hypothesis, we examined whether fumarate could rescue the survival ability of ASS1-KO cells after DNA damage. Indeed, we found that although adding fumarate did not alter the survival of cells expressing ASS1 following DNA damage, it decreased apoptosis and rescued survival in cancer cells with ASS1-KO (Fig. 3b and Extended Data Fig. 3h).

Hence, ASS1 and ASL participate in the nuclear generation of fumarate and increase survival following DNA damage.

### ASS1 is chromatin-bound

To further decipher the role of ASS1 in the nucleus, we performed an anti-ASS1 immunoprecipitation–MS analysis of the nuclear fraction in HCT116 colon cancer cells. Interestingly, pathway analysis revealed that several proteins interacting with ASS1 in the nucleus are related to chromatin remodeling, specifically to the SWI/SNF complex^25^ (Fig. 3c and Supplementary Table 1). We extracted nucleosomes from HCT116 colon cancer cells following Dox treatment to explore this connection further. We found that the nucleosome-bound fraction of ASS1 was elevated upon DNA damage (Fig. 3d). Similarly, we detected augmented chromatin binding of ASS1 upon DNA damage (Fig. 3e).

One core component of the SWI/SNF chromatin-remodeling complex is the SWI/SNF-related, matrix-associated, actin-dependent regulator of chromatin subfamily C member 1 (SMARCC1). SMARCC1, pulled down by Anti-ASS1 (Supplementary Table 3), participates in the dynamic epigenetic regulation of gene transcription, cell cycle progression and DDR^26,27^. Interestingly, SMARCC1 has been described to be regulated by succination, a fumarate-dependent post-translation modification^28,29^. Indeed, we found an interaction between ASS1 and SMARCC1 in the nucleus at basal conditions. This interaction significantly intensifies upon Dox treatment (Fig. 3f). Strikingly, we found that ASL also interacts with SMARCC1 and that this interaction significantly intensifies following Dox treatment and decreases with ASS1 loss (Fig. 3g). Therefore, we theorized that the role of ASS1 in the chromatin fraction might be related, at least partly, to its interaction with SMARCC1 and fumarate generation.

### ASS1 regulates SMARCC1 succination following DNA damage

**Fig. 4:**
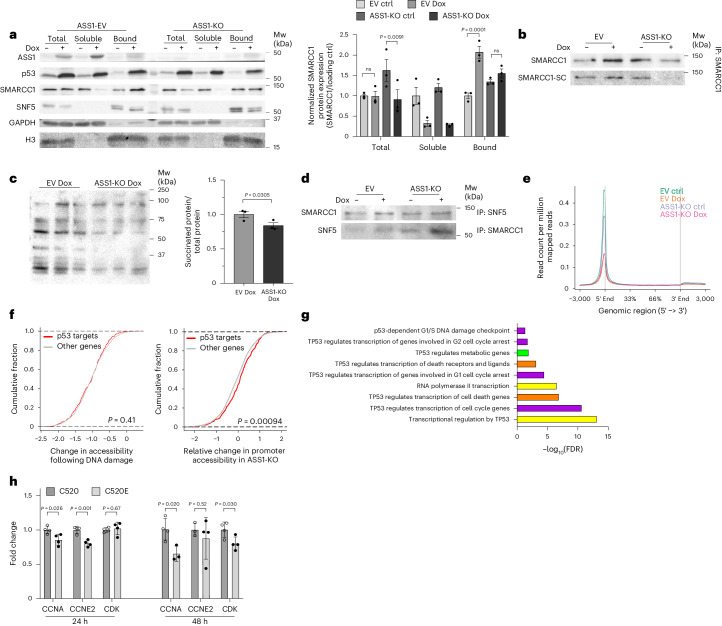
ASS1 regulates p53-related gene transcription following DNA damage via SMARCC1 succination. **a**, Control and Dox-treated HCT116 ASS1-EV (EV) and ASS1-KO cells were assayed for the total, soluble and chromatin-bound fraction and immunoblotted for ASS1, SMARCC1, SNF5 and p53. Markers: H3, nuclear; GADPH, cytoplasmic. *n* = 3 independent experiments. Right panel: quantification of total and soluble SMARCC1 levels relative to GAPDH, and nuclear SMARCC1 levels relative to H3. **b**, Immunoprecipitation with anti-SMARCC1 of the chromatin-bound fraction of control and Dox-treated HCT116 ASS1-EV (EV) and ASS1-KO cells and immunoblotted for SMARCC1 and SC. *n* = 3 independent experiments. **c**, The nuclear fraction of control and Dox-treated HCT116 ASS1-EV (EV) and ASS1-KO cells immunoblotted for general succination (SC). *n* = 3 biologically independent samples. **d**, Immunoprecipitation with anti-SMARCC1 or anti-SNF5 of the chromatin-bound fraction of control and Dox-treated HCT116 ASS1-EV (EV) and ASS1-KO immunoblotted for SNF5 or SMARCC1, respectively. *n* = 3 independent experiments. **e**, Mapping of read counts of promoter accessibility using ATAC–seq of control and Dox-treated HCT116 ASS1-EV (EV) and ASS1-KO cells. *n* = 3 biologically independent samples. **f**, Promoter accessibility of p53 DDR target genes using ATAC–seq of control and Dox-treated HCT116 ASS1-EV (left) and ASS1-KO cells (right). *n* = 3 biologically independent samples. **g**, Pathway enrichment analysis of p53 target genes significantly changing between Dox-treated HCT116 ASS1-KO and EV cells. Each bar shows the fold enrichment of a specific pathway. The bars are color-coded according to the different pathway types: yellow, transcription general; purple, cell cycle; orange, death; green, metabolism. *n* = 5 biologically independent samples. FDR, false discovery rate. **h**, Real-time PCR for p53 cell cycle genes performed on SKOV3 cells transfected with GFP, SMARCC1 C520 or SMARCC1 C520E plasmids for 24 h or 48 h. *n* = 3 biologically independent samples for (C520, *CCNE2*, 24 h), (C520E, *CCNA*, 48 h), (C520, *CCNE2*, 48 h); all others, *n* = 4 biologically independent samples. Data are represented as the mean ± s.e.m. ns, not significant. *P* values were determined by two-way ANOVA with Tukey’s multiple-comparison test (in **a**), Wilcoxon rank-sum test (in **f**) or unpaired two-tailed Student’s *t*-test (in **c** and **h**). Source data

**Extended Data Fig. 4:**
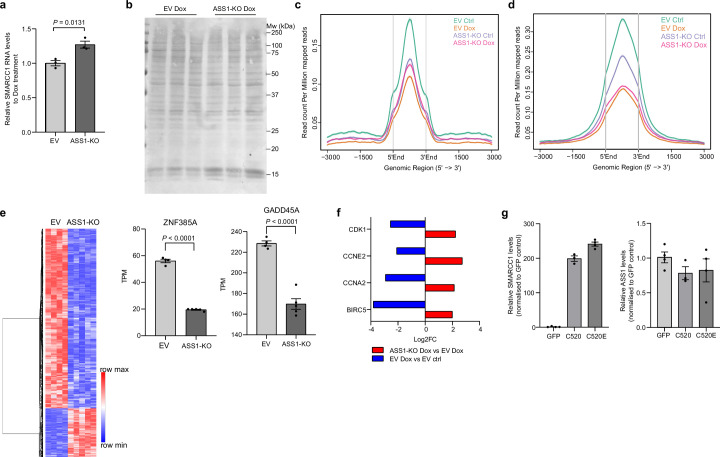
ASS1 regulates p53-related gene transcription following DNA damage via SMARCC1 succination. **a**, HCT116 ASS1-EV (EV) and ASS1-KO cells were incubated with (Dox) or without Dox (Ctrl) for 2 hrs, and incubated for 48 hrs. RNA was extracted and *SMARCC1* RNA levels were detected using quantitative real-time PCR. (*n* = 3 biologically independent samples) **b**, HCT116 ASS1-EV (EV) and ASS1-KO cells were incubated with Dox for 2 hrs, and after 48 hrs, fractionated and the nuclear fraction was immunoblotted for succination (SC). Prior to succinaton staining, the membrane was stained with Ponceau for total protein. (*n* = 3 biologically independent samples) **c**, Mapping of read counts of promoter accessibility using ATAC-Seq around peaks of SMARCC1 binding to chromatin in HeLa cells from the ENCODE project (ENCODE experiment ENCFF971JGA). **d**, Mapping of read counts of promoter accessibility using ATAC-Seq around peaks of H3K27ac enhancer-associated histone marks, when considering only intergenic peaks, as annotated by HOMER^59^, data from the ENCODE project (ENCODE experiment ENCSR661KMA). **e**, **Left panel:** Heat-map analysis of transcriptomic data comparing changes in gene expression between EV Ctrl and ASS1-KO Ctrl cells. (*n* = 5 biologically independent samples) **Right panels**: RNA expression levels of selected cell cycle and survival genes regulated by p53. (P-value: ZNF385A: <0.0001; GADD45A: <0.0001) (*n* = 5 biologically independent samples) **f**, RNA expression levels of p53-related genes are down-regulated in EV treated with Dox vs. untreated, while their expression is increased in ASS1 KO cells treated with Dox compared to treated EV. (*n* = 5 biologically independent samples) **g**, SKOV3 cells were transfected with GFP, SMARCC1 C520 or SMARCC1 C520E plasmids for 48 hrs, and subsequently real-time PCR for *SMARCC1* and *ASS1* was performed to verify transfection. (*n* = 4 biologically independent samples) Data are represented as the mean ± s.e.m. ns, not significant. *P* values were determined by unpaired two-tailed Student’s *t*-test (**a**, **e**, and **g**).

SMARCC1 succination destabilizes its interaction with SWI/SNF-related, matrix-associated, actin-dependent regulator of chromatin, subfamily B, member 5 (SNF5)^28^. SNF5 is an important SWI/SNF complex component that regulates gene transcription by affecting chromatin accessibility^28^. To investigate whether ASS1 is required to succinate SMARCC1, we first measured SMARCC1 levels in the chromatin-bound fraction following DNA damage in colon cancer cells without ASS1. We found that with ASS1 loss, total SMARCC1 levels decreased following Dox treatment, suggesting that ASS1 is required to maintain the protein levels of SMARCC1 (Fig. 4a). Additionally, although SMARCC1 nuclear levels increased following Dox addition, such an increase was not detected with ASS1 loss (Fig. 4a). Notably, following Dox, the RNA levels of *SMARCC1* were elevated in cells without ASS1, potentially supporting a compensatory mechanism following the reduced protein expression (Extended Data Fig. 4a). Following induction of DNA damage by Dox, SMARCC1 immunoprecipitation of the chromatin-bound fraction in cancer cells without ASS1 demonstrated decreased levels of SMARCC1 protein and lower levels of succinated SMARCC1 (Fig. 4b). Moreover, following ASS1 loss and Dox treatment, there was a decrease in the general nuclear protein succination (Fig. 4c and Extended Data Fig. 4b). These results suggest that nuclear ASS1 is required for nuclear succination following DNA damage and specifically for SMARCC1 succination.

We further tested the dependence of the SMARCC1–SNF interaction on ASS1 by immunoprecipitating the chromatin-bound fraction with anti-SNF5 and anti-SMARCC1. In ASS1-KO cells, upon Dox treatment, we detected augmented SMARCC1–SNF5 interaction, supporting the notion that this interaction may be inhibited by ASS1-dependent SMARCC1 succination (Fig. 4d). An expected outcome of disrupting the SMARCC1–SNF5 interaction by ASS1-dependent succination is decreased chromatin accessibility, leading to reduced gene transcription. We therefore performed an assay for transposase-accessible chromatin sequencing (ATAC–seq)^30^. Following Dox induction of DNA damage, ASS1-KO and parental colon cancer cells had a more significantly reduced accessibility over all promoters and, to a lesser degree, in enhancers (Fig. 4e and Extended Data Fig. 4c,d). Supporting our notion that the loss of ASS1 causes DNA damage, we found a baseline decrease in promoter accessibility in untreated ASS1-KO cells (Fig. 4f and Extended Data Fig. 4c). RNA-seq analysis demonstrated that following ASS1 loss, the transcription of multiple genes is decreased compared to WT and the p53-related genes regulating the cell cycle and survival^31,32^ are specifically and significantly affected (Fig. 4g, Extended Data Fig. 4e and Supplementary Tables 4 and 5). Despite the overall trend, ATAC–seq analysis showed that in ASS1-KO cells, p53-related DDR genes exhibited relatively less reduction in promoter accessibility (Fig. 4f). Correspondingly, zooming in on the RNA-seq analysis reveals that a subset of p53-regulated genes indeed have a higher expression in ASS1-KO cells treated with Dox compared to WT ASS1-expressing cells treated with Dox. By contrast, the expression of the same genes is downregulated in control ASS1-expressing Dox-treated colon cancer cells relative to untreated **(**Extended Data Fig. 4f and Table 6). These findings suggest that although there is a general decrease in transcription following DNA damage, ASS1 is probably necessary to restrict more specifically the transcription of several p53-regulated genes.

Finally, to confirm the mechanistic connection between SMARCC1 succination and the regulation of p53 genes following DNA damage, we used SKOV3 ovarian cancer cells that do not express SMARCC1 (ref. ^33^). We transfected SKOV3 cancer cells with either WT *SMARCC1* or with the *SMARCC1* carrying the C520E mutation, which has been shown to abrogate the ability of SNF5 to capture SMARCC1 (ref. ^28^). This mutation replicates the succination scenario following DNA damage, independent of ASS1. We found that indeed, cells carrying the *SMARCC1* C520E mutation demonstrate a significant decrease in the expression of p53-regulated cell cycle genes, which we found to be downregulated following DNA damage in colon cancer cells expressing ASS1 (Fig. 4h and Extended Data Fig. 4f,g).

**Fig. 5:**
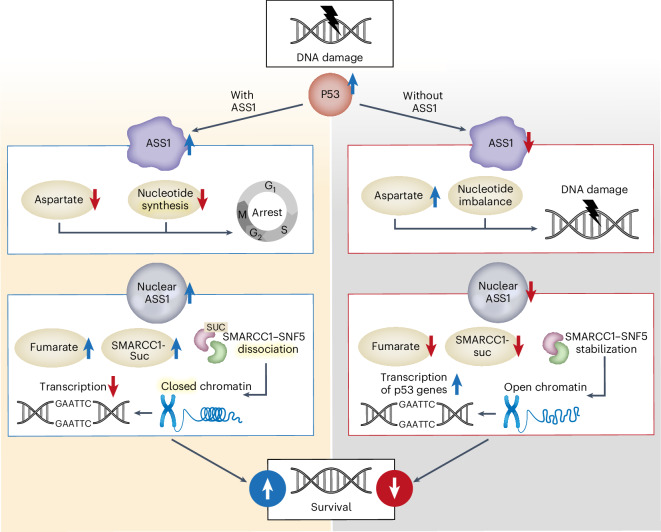
Suggested model for the requirement of ASS1 in the p53-response to DNA damage. In cells expressing ASS1, upon DNA damage, p53 levels are elevated, upregulating the expression of ASS1. ASS1 acts as a metabolic cell cycle checkpoint in the cytosol by regulating nucleotide levels. In the nucleus, ASS1 regulates fumarate levels needed for the succination of SMARCC1. Upon succination, there is a dissociation of SMARCC1 from SNF5, decreasing chromatin accessibility and p53-related transcription. In the absence of ASS1, there is an increase in cytosolic aspartate levels, leading to unregulated nucleotide synthesis and promoting DNA damage. In the nucleus, loss of ASS1 expression leads to increased SMARCC1–SNF complex, higher chromatin accessibility and transcription of p53 genes. Upwards and downward arrowheads indicate an increase or decrease, respectively. The scheme was created by Weizmann Graphics team.

In summary, ASS1 is regulated by p53 to maintain genome integrity metabolically; at basal state, ASS1 regulates nucleotide synthesis and cell cycle entrance, and hence its loss dysregulates nucleotide synthesis, promoting DNA damage and decreasing survival. Following DNA damage, cytosolic ASS1 is essential to decrease aspartate and nucleotide synthesis to pause the cell cycle. In the nucleus, ASS1 is necessary to generate fumarate for SMARCC1 succination, reducing promoter accessibility in general and, specifically, the transcription of p53-regulated cell cycle genes (Fig. 5).

## Discussion

This study demonstrates cytosolic and nuclear roles for ASS1, at least partly regulated by p53, integrating cellular amino acid metabolism with the DDR. Although ASS1 is required for similar functions in non-transformed and cancerous cells, its loss in cancer cells can provide survival benefits. We previously demonstrated in multiple cancers that ASS1 downregulation increases aspartate availability for pyrimidine synthesis, supporting proliferation and contributing to mutagenesis by generating a high pyrimidine-to-purine imbalance^4–6^. We now show that loss of ASS1 in colon cancer cells dysregulates nucleotide synthesis, enabling cell cycle progression despite DNA damage (Fig. 5). Recent studies highlight the importance of error-prone polymerase to cancer mutagenesis, supporting drug resistance and survival in changing environments^34^. Thus, ASS1 downregulation may further contribute to error-prone mutagenesis, increasing cancer adaptability potential and survival.

Notably, we further demonstrate localization of ASS1 to the nucleus, binding to the chromatin and regulating p53-gene transcription by increasing fumarate availability for SMARCC1 succination, restricting SMARCC1–SNF5 interaction and decreasing p53 cell cycle gene transcription (Fig. 5). Loss of ASS1 results in increased transcription of p53-related genes, demonstrating the requirement of ASS1 for fine-tuning repression of p53 regulated cell cycle genes following DNA damage, probably contributing to cell cycle progression. Although the mechanisms may vary, p53 repression of transcription usually does not involve direct binding of p53 to the DNA of repressed genes^35^. Here, we provide an additional mechanism for p53 inhibition of gene transcription by upregulating nuclear ASS1 levels and regulating chromatin opening. These findings add to the current literature on the SWI/SNF complex for p53-driven transcriptional regulation of cell cycle control^36^ and response to DNA damage^37^.

Moreover, our results align with recent literature showing that tricarboxylic acid cycle subnetworks operate in the nuclei in histones and nucleic acid modifications^38^, demonstrating the importance of compartmental tracing for the presence of critical metabolites at different and specific states. Specifically, our data showing fumarate generation in the nucleus by ASS1–ASL addresses an important question regarding the sources of nuclear fumarate^24^. It was previously revealed that fumarase is phosphorylated and recruited to DNA double-strand break regions by the DNA-dependent protein kinase complex for the local generation of fumarate from malate^24^, leading to the repair of the break by the non-homologous end-joining DNA-repair pathway and supporting cell survival^39^. In fact, congenital loss of fumarase results in a predisposition to hereditary leiomyomatosis and renal cell cancer syndrome, which has been demonstrated to be a DNA-repair deficiency syndrome^40,41^. We now show that as part of the response to DNA damage and fumarase generation of fumarate by functioning in reverse^24^, ASS1 and ASL work together to generate and increase the levels of nuclear fumarate. Notably, prolonged loss of ASS1 results in compensatory metabolic adaptations for supplying fumarate for succination, while during acute DNA damage caused by Dox, the necessity of ASS1 for succination is more prominent.

Maintaining genome integrity is vital to cell survival; all related metabolic processes, including nucleotide synthesis, are tightly regulated at multiple levels. Our results highlight cytosolic and nuclear roles for ASS1 as a metabolic guardian of genome integrity at physiological and pathological states. Yet for cancer, imperfect genome integrity enables adaptations and promotes survival. Translationally, our data imply that cancer patients and CTLN-I patients with ASS1 loss may be more sensitive to DNA-damaging agents. Broadly, our findings propose additional mechanisms by which the downregulation of ASS1 in cancers promotes carcinogenesis and survival.

## Methods

### Ethics and inclusion statement

The research included local researchers throughout the research process: study design, study implementation, data ownership, intellectual property and authorship of publications. Roles and responsibilities were agreed upon amongst collaborators ahead of the research.

### Cell lines

HCT116 WT, HCT116 p53 EV, HCT116 p53 KO and MC38 cells were kindly provided by Professor Moshe Oren, Department of Molecular Cell Biology, Weizmann Institute of Science. Normal healthy fibroblasts (ATCC PCS-201-012 lot no. 70015617, lot no. 80616177; Sigma-Aldrich, C-12302) were kindly provided by Professor Nicola Brunetti-Pierri. CTLN-I fibroblasts (GM01679) were purchased from Coriell Medical Institute. LS-174-T cells were kindly provided by Professor Deborah Fass, Department of Chemical and Structural Biology, Weizmann Institute of Science. SKOV3 cells were purchased from ATCC (cat. no. HTB-77, RRID:CVCL_0532). Cells were cultured using standard procedures in a 37 °C humidified incubator with 5% CO_2_ in DMEM (Biological Industries) (HCT116, MC38, SKOV3) or MEM (LS-174-T), supplemented with 10% heat-inactivated FBS (Gibco), 10% penicillin–streptomycin (Biological Industries) and 2 mM l-glutamine (Biological Industries). HCT116 colon cancer cells were cultured in a SILAC medium (Thermo Fisher Scientific) without arginine or in Plasmax without Arginine or without Arginine and Aspartate, prepared according to a previous publication^42^. All cells were routinely tested for mycoplasma using a Mycoplasma EZ-PCR test kit (Biological Industries).

### Dox treatment

DNA damage was induced using Dox. Cells were incubated with 2 μg ml^−1^ Dox (Sigma-Aldrich) for 2 h at 37 °C, after which the cells were washed and fresh media was added. Cells were collected for further downstream applications after 48 h.

### ASS1 CRISPR KO

Guide RNAs were designed to NM_000050.4, using Benchling (www.benchling.com)^43,44^ and CRISPOR (http://crispor.tefor.net)^45^, and should delete all isoforms of ASS1. Guide 1 (GGGGGCAGACACGTCGTGAG) targeted intron 1 and guide 2 (CGTCATTGCCTATCTGGTGA) targeted exon 3. The protocol was adapted from a previous publication^46^. Guide RNAs were inserted into pKLV-U6gRNA(BbsI)-PGKpuro2ABFP (Addgene, 50946). HCT116 WT cells were transfected with a plasmid containing guide 1, a plasmid containing guide 2 and pCas9-GFP (Addgene, 44719), according to the Lipofectamine 2000 (Invitrogen, 11668019) protocol, to generate ASS1-KO cells. EV cells were generated using pKLV-U6gRNA(BbsI)-PGKpuro2ABFP (Addgene, 50946) and pCas9-GFP plasmids (Addgene, 44719). Cells were sorted according to the fluorescence marker. Cells were checked for KO of ASS1 by a citrulline-arginine rescue assay using XTT, real-time quantitative PCR and western blot.

Primers for guide RNA insertion:

Guide 1 forward primer: CACCGGGGGCAGACACGTCGTGAGGT

Guide 1 reverse primer: TAAAACCTCACGACGTGTCTGCCCCC

Guide 2 forward primer: CACCCGTCATTGCCTATCTGGTGAGT

Guide 2 reverse primer: TAAAACTCACCAGATAGGCAATGACG

### Subcellular fractionation

Cells were grown to ~80% confluency and collected with trypsin. The fractionation protocol was adapted from a previous publication^47^. Cells were washed twice with cold PBS (1000×*g*, 3 min, 4 °C) and then resuspended in lysis buffer (10 mM HEPES pH 7.5, 10 mM KCl, 0.1 mM EDTA, 1 mM dithiothreitol (DTT), 0.5% NP-40), supplemented with protease and phosphatase inhibitor cocktails (Sigma-Aldrich) and incubated on ice for 20 mins with gentle shaking. After the incubation, cells were vortexed and centrifuged for 10 min at 12,000×*g* at 4 °C, the supernatant was collected as the cytoplasmic fraction and the pellet was washed twice more with the lysis buffer. The pellet was then resuspended in mitochondrial isolation buffer (250 mM sucrose, 20 mM HEPES, 10 mM KCl, 1.5 mM MgCl_2_, 1 mM EDTA, 1 mM EGTA, pH 7.4) supplemented with protease and phosphatase inhibitor cocktails (Sigma-Aldrich) and centrifuged for 10 min at 1,000×*g* at 4 °C. The supernatant was discarded and the pellet was washed a further three times with cold PBS (800×*g*, 10 min, 4 °C). The pellet was resuspended in nuclear lysis buffer (1.5 mM MgCl_2_, 0.2 mM EDTA, 20 mM HEPES, 0.5 M NaCl, 20% glycerol, 1% Triton X-100, pH 7.4) and incubated on ice for 30 min and then sonicated at 3 Å for ~7 s (two times). Samples were then centrifuged for 15 min at 16,000×*g*. The supernatant contained the enriched nuclear fraction.

The liver tissue protocol was adapted from a previous publication^48^. Liver samples were homogenized using a Teflon pestle and mortar and suspended in mitochondrial isolation buffer (250 mM sucrose, 20 mM HEPES, 10 mM KCl, 1.5 mM MgCl_2_, 1 mM EDTA, 1 mM EGTA, pH 7.4) supplemented with protease and phosphatase inhibitor cocktails (Sigma-Aldrich). Lysates were centrifuged at 1,000×*g* for 10 min at 4 °C to pellet the nuclei while mitochondrial and cytosolic fractions were contained within the supernatant. Pellets containing nuclei were washed three times with PBS and centrifuged at 800×*g* for 10 min. Pellets containing nuclei were resuspended in nuclear lysis buffer (1.5 mM MgCl_2_, 0.2 mM EDTA, 20 mM HEPES, 0.5 M NaCl, 20% glycerol, 1% Triton X-100, pH 7.4) and incubated on ice for 30 min and then sonicated at 3 Å for ~10 s (three times). Samples were then centrifuged for 15 min at 16,000×*g*. The supernatant contained the enriched nuclear fraction. Supernatants containing mitochondrial and cytosolic fractions were recentrifuged twice at 16,000×*g* for 20 min at 4 °C to pellet the mitochondria. The resulting supernatant was the enriched cytosolic fraction. The mitochondrial pellets were then resuspended in a fourfold dilution of mitochondrial isolation buffer and centrifuged at 16,000×*g* for 20 min at 4 °C twice. The mitochondrial pellets were resuspended in a onefold dilution of mitochondrial isolation buffer and then sonicated to yield the mitochondrial fraction.

### Western blot analysis

Total cell lysates were collected using RiPA (Sigma-Aldrich) and a 1% protease inhibitor cocktail (Calbiochem). The supernatant was collected after centrifugation, and protein concentration was measured using a BCA Protein Assay Kit (ThermoFisher Scientific). A total of 20 μg from each sample, under reducing conditions, was separated using electrophoresis on a 12% SDS-polyacrylamide gel. Proteins were then transferred to cellulose nitrate membranes (Tamar). Non-specific binding was blocked with 5% skim milk in TBST (10 mM Tris-HCl (pH 8.0), 150 mM NaCl, 0.1% Tween-20) for 45 min, shaking at room temperature (20–22 °C). Membranes were incubated overnight with antibodies against human ASS1 (ab124465; 1:1,000), ASL (ab97370; 1:1,000), tubulin (T5168, 1:3,000), GAPDH (ab128915; 1:5,000), p53 (sc-126; 1:1,000), p21 (ab109520; 1:10,000), lamin B1 (ab16048; 1:2,000), MEK1 + MEK2 (ab178876; 1:10,000), importin 7 (ab99273; 1:1,000), γH2AX (CST-9718S; 1:1,000), CPS1 (ab45956; 1:1,000), H3 (BLG-819414; 1:1,000), SMARCC1 (ab126180; 1:1,000), SNF5 (CST91735S; 1:1,000), 2SC (crb2005017e; 1:1,000), CPS1 (ab45956; 1:1,000) and vinculin (CST-13901S; 1:1,000). Detection of primary antibodies was done using Donkey Anti-Rabbit IgG H&L (HRP) preadsorbed (ab97085; 1:10,000) and Donkey Anti-Mouse IgG H&L (HRP) preadsorbed (ab98799; 1:10,000) and enhanced chemiluminescence western blotting detection reagents (Azure). Membranes were captured by Gel Doc XR+ (BioRad) and analyzed by ImageLab 5.1 software (BioRad) and ImageJ (NIH). Equal amounts of protein were loaded for the cytoplasmic and nuclear fractions. The band area was calculated by the intensity of the band. The obtained value was then divided by the value obtained from the loading control. The cytoplasmic ratio was calculated by measuring the intensity of the band and dividing it by the band intensity of the loading control (tubulin or GAPDH). Similarly, the nuclear ratio was calculated by measuring the intensity of the band and dividing it by the band intensity of the loading control (H3).

### Proximity ligation assay

Protein–protein interactions were detected with Duolink in situ PLA technology probes and reagents (Sigma-Aldrich), according to the manufacturer’s protocol. Cells were grown to ~60% confluency on coverslips in 12-well plates, fixed in 4% PFA for 20 min at room temperature and incubated with blocking solution (2% BSA in PBS) for 20 min at room temperature. Permeabilization was performed with PBS + 0.1% Triton X-100 for 3 min at room temperature. The samples were incubated with primary antibodies for 1 h at room temperature, followed by a 1 h incubation at 37 °C with the PLA probes (anti-mouse MINUS and anti-rabbit PLUS). Antibodies used for detection were diluted in blocking solution; ASS1 (ab124465; 1:100), importin 7 (ab99273; 1:100), ASL (sc-374353; 1:100) and SMARCC1 (ab126180; 1:100). The cells were washed three times for 5 min with buffer A (0.01 M Tris-HCl pH 7.4, 0.15 M NaCl and 0.05% Tween-20) and then the ligation step was performed for 30 min at 37 °C. After this step, the coverslips were washed with buffer A twice for 2 min and the amplification step was performed for 100 min at 37 °C. After two washes with buffer B (0.2 M Tris-HCl pH 7.5, 0.15 M NaCl), the coverslips were washed with PBS and mounted with mounting medium with DAPI–Aqueous, Fluoroshield (ab104139), stored at 4 °C overnight and imaged the following day. The PLA puncta, distinct fluorescent spots, were visualized by a confocal spinning disk microscope (Zeiss Observer AX10, Yokogawa CSU-X1). Nuclei were counter-stained with DAPI. Z-stack images were taken and the max intensity z-stack projection was used for image analysis. Image processing and quantification were performed with ImageJ.

### Gas chromatography–MS

Cells, after Dox treatment, were washed with ice-cold saline and lysed with a mixture of 50% methanol and water, with 2 μg ml^−1^ ribitol added as an internal standard. Cells were quickly scraped followed by three freeze–thaw cycles in liquid nitrogen. The insoluble material was pelleted and the supernatant was collected for subsequent gas chromatography–MS analysis. Samples were dried overnight using a vacuumed lyophilizer. Dried samples were treated with 40 μl of a methoxyamine hydrochloride solution (20 mg ml^−1^ in pyridine) at 37 °C for 90 min while shaking, followed by incubation with 70 μl 1 N,O-bis(trimethylsilyl)trifluoroacetamide (Sigma-Aldrich) at 37 °C for an additional 30 min.

Gas chromatography–MS analysis used a gas chromatograph (7820AN, Agilent Technologies) interfaced with a mass spectrometer (5975 Agilent Technologies). An HP-5ms capillary column 30 m × 250 μm × 0.25 μm (19091S-433, Agilent Technologies) was used. Helium carrier gas was maintained at a constant flow rate of 1.0 ml min^−1^. The gas chromatogrph column temperature was programmed from 70 to 150 °C with a ramp of 4 °C min^–1^, 250–215 °C with a ramp of 9 °C min^–1^, 215–300 °C with a ramp of 25 °C min^−1^ and then maintained at 300 °C for an additional 5 min. The MS was by electron impact ionization and operated in full-scan mode from *m*/*z* = 30–500. The inlet and MS transfer line temperatures were maintained at 280 °C and the ion source temperature was 250 °C. Sample injection (1 μl) was in splitless mode. Total metabolite levels were normalized to ribitol and to the protein levels of each sample, and metabolite ratios were calculated using the area under the curve ratios per sample.

### Nucleotide analysis

#### Materials

Sodium salts of nucleoside monophosphates and deoxynucleoside triphosphates were obtained from Sigma-Aldrich. Labeled adenosine-^15^N_5_ 5′-monophosphate disodium salt and adenosine-^13^C_10_ 5′-triphosphate disodium salt were used as internal standards.

#### Sample preparation

The collected cells were extracted and purified on polymeric weak anion columns (Strata-XL-AW 100 μm (30 mg ml^−1^, Phenomenex)) and dried as described previously^4^. The obtained residues were re-dissolved in 50 μl of 50% aqueous acetonitrile before liquid chromatography–MS (LC–MS) analysis.

#### LC–MS/MS analysis

An Acquity I-class ultra-performance liquid chromatography (UPLC) system (Waters) and Xevo TQ-S triple quadrupole mass spectrometer (Waters) equipped with an electrospray ion source (ESI) and operated in positive ion mode was used for the analysis of nucleoside monophosphates. MassLynx and TargetLynx software (version 4.2, Waters) were applied for the acquisition and analysis of data. Chromatographic separation was performed on a 150 mm × 2.1 mm internal diameter, 1.7 μm Atlantis Premier BEH-Z-HILIC column equipped with a VanGuard filter. Mobile phases A (20 mM ammonium carbonate:acetonitrile (80:20) (pH 9.2) and B (acetonitrile) were used at a flow rate of 0.3 ml min^–1^ and column temperature 25 °C for gradient elution as follows: 0–0.8 min at 80% B, then 0.8–5.6 min a linear decrease to 25% B, 5.6–6.0 min held at 25% B and 6.0–6.5 min back to 80% B. Then the column was re-equilibrated for an additional 2.5 min. Samples were kept at 8 °C and automatically injected in a volume of 1 μl. For MS, argon was used as the collision gas with a flow of 0.10 ml min^–1^. The capillary voltage was set to 2.50 kV, source temperature 150 °C, desolvation temperature 400 °C, cone gas flow 150 l h^–1^ and desolvation gas flow 800 l h^–1^. Multiple-reaction monitoring (MRM) parameters for nucleoside monophosphates are listed in supplementary Table 3 in a previous publication^4^. MRM parameters (collision energy (CE)) for deoxynucleoside triphosphates were as follows: 468.0 > 112.2 and 468.0 > 192.1 *m*/*z* (CE = 12 eV for both) for dCTP, 483 > 81 *m*/*z* (CE = 27 eV) for dTTP, 492.1 > 81.1 and 492.1 > 136.3 *m*/*z* (CE = 24 and 16 eV, respectively) for dATP and 507.9 > 152.1 *m*/*z* (CE = 30 eV) for dGTP. Internal standards: 353.1 > 141.1 *m*/*z* (CE = 20 eV) for ^15^N_5_-AMP, and 518.1 > 141.1 and 518.1 > 420.1 *m*/*z* (CE = 35 and 18 eV, respectively) for ^13^C_10_-ATP.

Nucleotide concentration was calculated using a standard curve of the relevant nucleotide concentrations in each sample. Standard curves included increasing concentration of all measured nucleotides ranging from 0–10 µg ml^−1^ that were positioned at the beginning and end of each run. All calculated values for the different nucleotides in each sample fell within the standard curve range.

### Nuclear isolation and tracing

Nuclear extracts were prepared as previously described^47^. For cell lines, after washing with PBS, 1–1.5 ×10^7^ cells were suspended in cell lysis buffer (10 mM HEPES pH 7.5, 10 mM KCl, 0.1 mM EDTA, 1 mM DTT, 0.5% NP-40 and protease–phosphatase inhibitor cocktail) for 20 min on ice with intermittent gentle mixing. At the end of incubation, tubes were vortexed and nuclei were pelleted at 12,000×*g* for 10 min at 4 °C by centrifugation followed by two more washes with the lysis buffer. The nuclei pellet was further resuspended in ice-cold fractionation buffer (2 M sucrose, 1 mM MgCl_2_ and 10 mM Tris-HCL pH 7.4), mixed well and then centrifuged at 16,000×*g* for 30 min at 4 °C. After removal of the supernatant, the nuclei were washed twice with cell lysis buffer. Nuclei were resuspended in 100 µl of respiration buffer (125 mM KCl, 2 mM MgCl_2_, 2.5 mM KH_2_PO_4_, 20 mM HEPES pH 7.2, 1 mM ATP, 0.01 mM ADP, 1 mM NAD^+^, 0.01 mM NADH) supplemented with 2 mM of ^13^C_4_-aspartate (Sigma-Aldrich, 604852) and 2 mM citrulline for 4 h at 37 °C with periodic mixing. An equal amount of 100% MeOH spiked with ribitol as an internal standard was added to the respiration buffer at the end of the incubation period. Samples were centrifuged and the supernatant was collected for subsequent LC–MS analysis. Samples were dried using a vacuumed SpeedVac followed by lyophilizer overnight. The samples were resuspended in 70 µl MeOH and 1 µl was injected into the Q Exactive Plus Hybrid Quadrupole-Orbitrap (Thermo Fisher Scientific). Data processing was done with TraceFinder. The areas of the peaks were corrected using Isocor^49^ to account for naturally occurring isotopes, and the ratio of the area under the curve for M + 4 of fumarate to the M + 4 of aspartate was used to calculate the enrichment in production of labeled fumarate. The peak we identified for fumarate can additionally result from interference from malate. This can be attributed to the fact that malate loses one molecule of H_2_O during ionization and becomes identical to fumarate. Hence, our results likely reflect the combined interference of fumarate with malate.

### Nuclei extraction and separation from mice liver

ASS1^Flox/Flox^ and ASS1 Alb-cre mice were anesthetized. Livers were dissected and washed with cold PBS. Nuclei were extracted from 100 mg of liver tissue using nuclei extraction buffer (Miltenyi Biotech, cat. no. 130-128-024) and purified using Anti-Nucleus MicroBeads (Miltenyi Biotech, cat. no. 130-132-997) according to Miltenyi Biotech products protocols. In brief, 100 mg of liver tissue was dissociated and lysed in ice-cold Nuclei Extraction Buffer containing protease inhibitors cocktail (Sigma-Aldrich, cat. no. P8340) in gentleMACS C Tubes (Miltenyi Biotech, cat. no. 130-093-237) using the 4C_nuclei_1 program in gentleMACS Octo Dissociator (Miltenyi Biotech) in cooling conditions. The lysates were filtered with a 70 µm strainer and centrifuged at 300×*g* at 4 °C for 5 min. The pellet was resuspended in ice-cold Anti-Nucleus MicroBeads separation buffer (containing 0.04% BSA and Nuclei Extraction Buffer diluted 1:7 in PBS, pH 7.2) and filtered again with a 40 µm strainer. The flow through containing the nuclei was kept on ice and the nuclei concentration was determined. For nuclei enrichment, approximately 6 × 10^6^ nuclei per sample were incubated with Anti-Nucleus MicroBeads for 15 min in a cold room (2–8 °C) and purified or separated on LS Columns (Miltenyi Biotech, cat. no. 130-042-401). Purified nuclei were centrifuged at 300×*g* at 4 °C for 5 min and resuspended with 250 µl of respiration buffer containing ^13^C_4_-aspartate for 3.5 h at 37 °C with periodic mixing. An equal amount of 100% MeOH was added to the respiration buffer at the end of the incubation period. Samples were centrifuged and the supernatant was collected for subsequent LC–MS analysis. Lyophilized samples were diluted in 120 µl MeOH and distilled deionized water (DDW) 1:1 (v/v), vortexed, sonicated for 10 min and centrifuged at 18,407×*g* at 4 °C for 10 min. Derivatization of fumaric acid and malic acid with 3-NPH was performed according to Han et al.^50^, with some modifications. To 40 µl of the sample was added 10 µl of 200 mM 3-NPH in ACN:DDW 1:1 (3-nitrophenylhydrazine hydrochloride; Sigma-Aldrich, N21804-5G) and 10 µl of 120 mM EDC in ACN:DDW 1:1 containing 6% pyridine (N(3-dimethylaminopropyl)-N’-ethylcarbodiimide hydrochloride; Sigma-Aldrich, 03449-1G), followed by incubation at 40 °C for 30 min at 94×*g*. After that, 10 µl of acetone was added and it was incubated for 5 min at 40 °C at 94×*g*, then 55 µl of 10% ACN in DDW was added, sonicated for 10 min and centrifuged at 18,407×*g* at 4 °C for 10 min and then transferred to vials.

Malic acid and fumaric acid were measured by UPLC-ESI-MS/MS equipped with an Acquity UPLC I-class system (Waters). The MS detector (Waters Xevo TQ-XS) was equipped with ane ESI source. The measurement was performed in negative ionization modes using MRM. MS parameters were as follows: the source and desolvation temperatures were maintained at 150 °C and 600 °C, respectively; the capillary voltage was set to 1.0 kV; nitrogen was used as desolvation gas and cone gas at the flow rate of 1,000 l h^−1^ and 150 l h^−1^, respectively. MRM transitions for fumaric acid and malic acid were optimized by direct injection. MRM transitions for fumaric acid, malic acid and their isotopologues are summarized in Supplementary Table 4.

Chromatographic separation was performed on an Acquity UPLC HSS T3 column (1.0 × 100 mm, internal diameter, 1.8 μm) (Waters). Mobile phase A consisted of 0.03% (v/v) formic acid in water (UPLC grade) and mobile phase B consisted of 0.03% (v/v) formic acid in acetonitrile. The column was maintained at 40 °C and the flow rate of the mobile phase was 0.3 ml min^−1^. Mobile phase B was run for 1.5 min at 5%, then it was gradually increased to 73% over 35 min, then gradually increased to 90% over 35.5 min, and then set to 5% for 39 min and run at 5% for 45 min. The injection volume was 1 µl.

### Nucleosome isolation

Cells were washed twice with buffer B (PBS, 1:100 protease inhibitors, 0.05% IGEPAL (NP-40)) and resuspended in 1× lysis buffer containing 1:1,000 Micrococcal Nuclease Solution (ThermoFisher). Cells were incubated at 37 °C for 20 min with shaking, based on previous calibration in this system. The reaction was stopped using 20 mM EGTA and the samples were prepared for western blot analysis (2× lysis buffer: 100 mM Tris-HCl, 300 mM NaCl, 2% Triton X-100, 0.2% sodium deoxycholate, 10 mM CaCl_2_).

### Chromatin-bound assay

Cells were grown to ~80% confluency, collected with trypsin and washed twice with ice-cold PBS. Cells were resuspended in 1,000 µl of ice-cold PBS. Then 200 µl of cells was placed into a fresh tube labeled ‘Total’. The remaining cells were pelleted and resuspended in buffer A (100 mM NaCl, 300 mM sucrose, 2 mM MgCl_2_, 20 mM PIPES pH 6.9, 1 mM EGTA, 0.2% Triton X-100) and rotated at a low speed for 10 min at 4 °C. Samples were centrifuged at 845×*g* for 5 min in a cold centrifuge. The supernatant was moved to a tube labeled ‘Soluble’. The pellet was washed twice with buffer A and the supernatant was discarded. The pellet was resuspended in 60 µl of buffer B (50 mM NaCl, 300 mM sucrose, 2 mM MgCl_2_, 20 mM PIPES pH 6.9, 1 mM EGTA) with DNase (37 µl ml^−1^, Qiagen) and incubated for 45 min at 37 °C with shaking. An additional 240 µl of buffer B was added to the bound fraction.

### Chromatin-bound preparation for immunoprecipitation

Cells were grown to ~80% confluency, collected with trypsin and washed twice with ice-cold PBS. Cells were resuspended in 1,000 µl of ice-cold PBS. The remaining cells were pelleted and resuspended in buffer A (100 mM NaCl, 300 mM sucrose, 2 mM MgCl_2_, 20 mM PIPES pH 6.9, 1 mM EGTA, 0.2% Triton X-100) and rotated at a low speed for 10 min at 4 °C. Samples were centrifuged at 845×*g* for 5 min in a cold centrifuge. The supernatant was moved to a tube labeled ‘Soluble’. After washing twice with buffer A, the pellet was resuspended in 1× lysis buffer containing 1:1,000 Micrococcal Nuclease Solution (ThermoFisher). Cells were incubated at 37 °C for 20 min with shaking, based on previous calibration in this system. The reaction was stopped using 20 mM EGTA and centrifuged for 10 min at 4 °C at maximum speed. Samples were then used in immunoprecipitation.

### Immunoprecipitation

Dynabeads Protein G (ThermoFisher) were used for immunoprecipitation. The beads were prepared according to the protocol provided. Beads were bound with either SMARCC1, ASS1 or IgG antibody. Samples, either the chromatin-bound, the cytoplasmic fraction or the nuclear fraction, were incubated with antibody-bound beads overnight. The beads were resuspended in elution buffer and loaded for western blot analysis.

### Immunoprecipitation–MS-based proteomic analysis

Cells were grown to ~80% confluency and collected with trypsin. Nuclear and cytoplasmic fractions were separated using the NE-PER Nuclear Extraction Kit (Thermo Scientific) according to the manufacturer’s protocol, and immunoprecipitation was performed for the cytoplasmic and nuclear fractions. Protein levels for each sample were measured using BCA (ThermoFisher), and equal amounts of protein were incubated with Dynabeads Protein G (ThermoFisher). The beads, after overnight incubation, were resuspended in Tris-SDS buffer (50 mM Tris, pH 7.5) and the supernatant was taken for MS.

#### Sample preparation

The eluted samples were reduced with 5 mM DTT and alkylated with 10 mM iodoacetamide in the dark. Each sample was loaded onto S-Trap microcolumns (Protifi) according to the manufacturer’s instructions^51^. In brief, after loading, samples were washed with 90:10% methanol:1 M ammonium bicarbonate. Samples were then digested with 400 ng trypsin for 1.5 h at 47 °C. The digested peptides were eluted using 50 mM ammonium bicarbonate; trypsin was added to this fraction and incubated overnight at 37 °C. Two more elutions were made using 0.2% formic acid and 0.2% formic acid in 50% acetonitrile. The three elutions were pooled together and vacuum-centrifuged to dry. Samples were kept at −20 °C until analysis.

#### Liquid chromatography

ULC–MS-grade solvents were used for all chromatographic steps. Each sample was loaded using splitless nanoUPLC (10 kpsi nanoAcquity; Waters). The mobile phase contained (1) H_2_O + 0.1% formic acid and (2) acetonitrile + 0.1% formic acid. Desalting of the samples was performed online using a reversed-phase Symmetry C18 trapping column (180 µm internal diameter, 20 mm length, 5 µm particle size; Waters). The peptides were then separated using a T3 HSS nano-column (75 µm internal diameter, 250 mm length, 1.8 µm particle size; Waters) at 0.35 µl min^−1^. Peptides were eluted from the column into the mass spectrometer using the following gradients: 4–33% B in 50 min, 33–90% B in 5 min, maintained at 90% for 5 min and then back to initial conditions.

#### MS

The nanoUPLC was coupled online through a nanoESI emitter (10 μm tip; New Objective) to a quadrupole orbitrap mass spectrometer (Q Exactive HFX; Thermo Scientific) using a FlexIon nanospray apparatus (Proxeon). Data were acquired in data-dependent acquisition mode, using a Top10 method. MS1 resolution was set to 120,000 (at *m*/*z* 200), a mass range of 375–1650 *m*/*z*, AGC of 3e6 and maximum injection time of 50 ms. MS2 resolution was set to 15,000 (at *m*/*z* 200), quadrupole isolation 1.7 *m*/*z*, AGC of 1e5, dynamic exclusion of 20 s and maximum injection time of 60 ms.

#### Data processing and analysis

Raw data were processed with MaxQuant v.1.6.6.0 (ref. ^52^). Data were analyzed with the Andromeda search engine against the human proteome database (www.uniprot.com) and appended with common laboratory protein contaminants, with the following modifications: carbamidomethylation of C as a fixed modification, and oxidation of M, deamidation of N or Q and amino-terminal acetylation as variable modifications. Protein quantification was based on unique peptides only, the minimal peptide ratio count was set to 1 and match between runs was enabled. The rest of the parameters were kept as default. The ProteinGroups file was used for further calculations using Perseus v.1.6.2.3. Decoy hits were filtered out, as well as proteins that were identified on the basis of a modified peptide only. The LFQ intensities were log transformed, and only proteins that had at least 50% valid values in at least one experimental group were retained. The remaining missing values were imputed. Student’s *t*-tests were performed based on the LFQ intensities, and the fold change (ratio) between the corresponding samples was calculated.

### ImageStream

For cell cycle analysis, cells were fixed with Cyto-Fast Fix/Perm buffer (Biolegend, 426803), and DAPI (422801, 1:500) was used to stain the nucleus. For γH2AX staining, cells were fixed with Cyto-Fast Fix/Perm buffer (Biolegend, 426803) and stained with γH2AX (CST-9718S; 1:100) overnight. After washing, cells were incubated with the Alexa Fluor 488 antibody (711-546-152; 1:500) for 1 h. DAPI (422801, 1:500) was used to stain the nucleus. After washing, cells were resuspended in 100 μl of PBS and collected using ImageStream; the analysis was performed on the IDEAS software. Images were acquired by ImageStreamX mark II (Amnis, Part of Luminex) using a ×60 lens (N.A., 0.9). At least 10^4^ cells were collected from each sample. The lasers used were 405 nm (120 mW), 642 nm (150 mW) and 785 nm (2 mW). Channels used were 1 (bright-field), 7 (DAPI), 9 (bright-field of the second camera), 11 (γH2AX) and 12 (side scatter). Images were analyzed using IDEAS v.6.3 software (Amnis, Part of Luminex). Live cells were gated according to their signal of the DAPI staining (Channel 7) (gating strategy). Cell doublets were then gated according to the area versus aspect ratio (the ratio between the minor axis and the major axis of a best-fit ellipse for the nuclear object) of the bright-field image. Focused cells were selected using the Gradient RMS feature (measures the sharpness quality of an image by detecting large changes of pixel values in the image, computed using the average gradient of a pixel normalized for variations in intensity levels). The γH2AX staining was quantified using the mean pixel feature (the mean of the background-subtracted pixels contained in the input mask, computed as intensity or number of pixels), to compensate for variability in cell size. The cell cycle was calculated using the DAPI staining (Channel 7).

### Flow Annexin V—apoptosis detection

HCT116 cells were plated at 60% confluency and grown for 24 h in full DMEM. Dox treatment was performed as previously mentioned in Plasmax medium without arginine and asparate. Fumarate rescue was performed by adding fresh media with 1 mM fumarate (Alfa Aesar) in DMSO (final concentration, 0.4%) or an equal concentration of DMSO after a 2 h incubation with 1 μg ml^−1^ Dox. Following 48 h incubation, cells were trypsinized, washed with PBS and stained with 400 nM Apotracker green (Biolegend, 427402) and DAPI (Biolegend, 422801) to detect apoptotic and dead cells. Data were acquired on CytoFLEX flow cytometer (Beckman Coulter) and analyzed with FlowJo software (Tree Star). The early and the late apoptotic gated cells were combined to calculate the percent of apoptotic cells per sample.

### Comet assay

The assay was conducted according to the comet assay kit (Abcam) protocol. In brief, 35 μl of suspended cells (1 × 10^6^ ml^−1^) was mixed with 150 μl agarose and 35 μl of this mixture was added to each agar-precoated slide. After incubation, the slides were immersed in pre-chilled lysis buffer for 1 h and replaced with a pre-chilled alkaline solution (300 mM NaOH, 1 mM EDTA-Na^2^, pH 13) for 30 min, all at 4 °C and in the dark. Then the slides underwent electrophoresis in TBE buffer for 20 min at 35 volts (1 V cm^−1^). After the run, the slides were dried, immersed twice with pre-chilled deionized water for 2 min, dehydrated in cold 70% ethanol for 5 min, stained with Vista Green DNA Dye (from the kit) at room temperature for 15 min and analyzed using a confocal microscope (FITC filter) (Zeiss LSM 880, EC Plan-Neofluar ×10/0.30 M27). Quantification was performed using the software Comet Score to determine the average tail DNA percentage.

### RNA-seq

RNA was extracted from EV and ASS1-KO cells with and without Dox induction using the Qiagen RNAeasy kit. Each set contained five biological repeats. The integrity of the RNA was assessed using TapeStation, and only samples with an RNI score of >8 were further processed. Library generation and RNA-seq were performed at NCI (NAS: NAS:CS031757) using the NovaSeq 6000 (Illumina, 20012850) sequencing system with the SP reagent kit (Illumina). Paired-end sequencing was performed with a read length of 150.

### Transcriptomic analysis

#### Data analysis

Differential expression was performed using DeSeq2 results function, with a log fold change threshold = 0.25 and AllHypothesis = ‘greaterAbs’. The Benjamini–Hochberg procedure corrected *P* values. Significant genes were chosen if they had adjusted *P* < 0.01. Differential expression was calculated by either comparing EV treated with Dox to ASS1-KO treated with Dox or comparing control EV samples to control ASS1-KO samples. Expression values were normalized by the DESeq2 DESeq function. Next, each value was subtracted by the mean of its gene expression in all samples.

#### Enrichment analysis

To evaluate pathways enriched in differentially expressed genes, we used the REACTOME pathway database.

#### p53 targets analysis

P53 target genes were chosen according to the census in a previous publication^53^. For the comparisons between ASS1 Dox versus EV Dox and EV Dox versus EV control, we detected the genes with significant log_2_ fold change in both comparisons.

### ATAC–seq

ATAC–seq was performed as previously described^54^ with minor adjustments for application to HCT116 cells. A total of 50,000 cells per sample were used. Libraries were sequenced with paired-end sequencing on a Novaseq 6000 machine. Three replicates were combined for all the analyses in the four groups (EV control, EV Dox, ASS1-KO control, ASS1-KO Dox). ATAC–seq reads were mapped to the human genome hg38 assembly using bowtie2 (ref. ^55^) and extended and normalized for overall sequencing depth using MACS2 (ref. ^56^). A metagene of the gene converge by ATAC–seq reads was prepared relative to the MANE gene annotations^57^. Genes were separated into promoter and gene body regions as previously described^58^, and HOMER^59^ was used to compute coverage in promoter regions and to compare conditions using DESseq2 (ref. ^60^). p53 target genes were taken from a previous publication^53^. *P* values for comparisons between gene groups were computed using the Wilcoxon rank-sum test.

### Bioinformatics analysis for p53 binding sites on ASL promoter

A total of 1,100 bp surrounding the ASL transcription start site (GRCh38 chr7:66,075,357–366,076,456) were taken from the UCSC genome browser (http://genome.ucsc.edu)^61^. The tracks shown were ReMap Altas of Regulatory Regions filtered for TP53, TP63 and TP73 binding sites (only TP53 was found)^62^, MatInspector (Genomatix Genome Analyzer)^63^ predicted p53 binding sites and RefSeq mRNAs. A binding site in HCT116 can be found in the proximal promoter, which overlaps a predicted binding site as well as binding peaks found in other cell lines. MatInspector analysis was performed on the genomic DNA imported into a local installation of the Genomatix Genome Analyzer, and hits to the p53 binding site, V$P53, were displayed in the UCSC genome browser as a custom track.

### Mice

Animal experiments were approved by the Weizmann Institute Animal Care and Use Committee following US National Institute of Health, European Commission and Israeli guidelines (IACUC 00260123-2). The authorization number from the Italian Ministry of Health is 413/2021-PR, project number CE571.64. ASL^neo/neo^ (B6.129S7-Asltm1Brle/J), ASS1^Flox/Flox^ (C57BL/6) and ASS1 Alb-cre (C57BL/6) mice were used. Care practices for mice included sterile conditions with sterile supplies. Mice were housed in individually ventilated cages, given ad libitum access to water and standard mouse chow with 12 hr light and dark cycles at 20–24 °C and 30–70% relative humidity.

### Short interfering RNA transfection

HCT116 WT cells seeded at roughly 60% confluency in DMEM. Scrambled short interfering RNA (siRNA) (5 nmol) or targeted siRNA (5 nmol) (Dharmacon ON-TARGETplus SMARTpool siRNA) in Opti-MEM reduced-serum medium (Thermo Fisher Scientific) was transfected at a final concentration of 25 nM with INTERFERin (Polyplus). After 6 h, the medium was changed to DMEM and incubated for 24 h before following the Dox induction protocol.

### Transient transfection

Plasmids encoding MYC-tagged SMARCC1, MYC-tagged SMARCC1 (C520E) and MYC-tagged GFP were a gift from J. Meier (Center for Cancer Research, NCI)^28^. SKOV3 cells were plated in 48-well plates (0.9 × 10^5^ cells per well), and concurrently, transient transfection of these plasmids (250 ng) was performed using Lipofectamine 2000 (Invitrogen) according to the manufacturer’s instructions. Expression was carried out by incubating the cells for 24–48 h at 37 °C under 5% CO_2_ atmosphere, after which they were collected for RNA extraction.

### Quantitative real-time PCR

RNA was extracted from cells using RNeasy Mini Kit (Qiagen) or Direct-zol RNA Miniprep Plus Kit (Zymo Research). cDNA was synthesized from 1 μg RNA using the qScript cDNA Synthesis Kit (Quanta). Detection on cDNAs was performed using SYBR Green FastMix Perfect CT (Quantabio) with the required primers (see qPCR primer list; Supplementary Table 7). Expression of genes of interest was normalized to the expression of the housekeeping gene human *Hprt* or to human *GAPDH*.

### Immunofluorescence

HCT116 cells were plated at 60% confluency on sterile coverslips in a 12-well plate for 24 h. The next day, cells were washed with PBS twice and fixated with 4% PFA (Santa Cruz Biotechnology) for 10 min. Cells were washed again and incubated with 0.1% Triton X-100 for 10 min at room temperature. Cells were washed and blocked with blocking solution (22.5 mg ml^−1^ glycine, 1% BSA, PBS-T) for 30 min at room temperature in a humidity assay. Cells were incubated with diluted primary antibodies in PBS-T (ASS1, 1:100; ASL, 1:100) overnight at 4 °C. The following day, cells were washed three times with PBS and then incubated with diluted secondary antibody in PBS-T (Alexa Fluor 594, 1:100) for 1 h at room temperature in a humidity chamber. Cells were washed three times with PBS and mounted with Fluoroshield Mounting Medium With DAPI (Abcam) on cover slides. Nuclei were counter-stained with DAPI. Slides were incubated overnight at 4 °C and imaged the following day using a confocal spinning disk microscope (Zeiss Observer AX10, Yokogawa CSU-X1). Image processing was performed using ImageJ.

### Cell proliferation assay

Cell proliferation was measured using a cell proliferation (XTT kit) (Biological Industries) following the manufacturer’s instructions and the absorbance was measured with Biotek Cytation 5 (Agilent). For CRISPR KO validation, HCT116 cells were plated at 60% confluency in a 96-well plate and grown for 24 h in full DMEM. The next day, the media was changed to arginine-free DMEM (day 0). Arginine and citrulline were added after 72 h to the respective wells. The absorbance was measured on day 5. For fumarate rescue, HCT116 cells were plated at 60% confluency and grown for 24 h in full DMEM. The following day, cells underwent Dox induction (2 μg ml^−1^) for 2 h (day 0). After 2 h, fresh media with 1 mM fumarate (Alfa Aesar) was added and absorbance was measured after a further 48 h.

### Statistics and reproducibility

Unless otherwise specified, all statistical analyses were performed using one-way ANOVA, or Student’s *t*-test or Wilcoxon rank-sum test of multiple or two groups, with Dunnett’s correction when required. The sample size was chosen in advance based on common practice of the described experiment and is mentioned for each experiment. When samples were distributed non-normally, Mann–Whitney analysis was performed. Data distribution was assumed to be normal but was not formally tested. Statistical tests were performed using GraphPad Prism v.8. No statistical method was used to predetermine sample size and no data were excluded from the analyses. The experiments were not randomized and the investigators were not blinded to allocation during experiments and outcome assessment. All error bars represent s.e.m. *P* ≤ 0.05 was considered significant in all analyses. Image analysis was performed using ImageJ v.1.54i. Images generated for Extended Data Fig. 1a and Extended Data Fig. 3g were created by the authors using licensed BioRender or PowerPoint tools, and not a third party.

### Reporting summary

Further information on research design is available in the Nature Portfolio Reporting Summary linked to this article.

### Supplementary information

Reporting Summary

Supplementary Data 1 Numerical source data for Supplementary Figures.

Supplementary Data 2 Uncropped scans for Supplementary Figures.

Supplementary Data 3 Live cells were gated according to their signal of the DAPI staining (Channel 7). Cell doublets were then gated according to the area vs aspect ratio (the ratio between the minor axis and the major axis of a best-fit ellipse for the nuclear object) of the bright-field image. Focused cells were selected using the Gradient RMS feature (measures the sharpness quality of an image by detecting large changes of pixel values in the image, computed using the average gradient of a pixel normalized for variations in intensity levels).

Supplementary Tables 1–7 Supplementary Tables.

### Source data

Source Data Fig. 1 Statistical source data.

Source Data Fig. 2 Statistical source data.

Source Data Fig. 3 Statistical source data.

Source Data Fig. 4 Statistical source data.

Source Data Fig. 1 Full-length, unprocessed gels or blots.

Source Data Fig. 2 Full-length, unprocessed gels or blots.

Source Data Fig. 3 Full-length, unprocessed gels or blots.

Source Data Fig. 4 Full-length, unprocessed gels or blots.

## Data Availability

All raw data are available in the Supplementary Information. RNA sequencing and mass spectrometry data can be found on Dryad at https://datadryad.org/stash/share/_SN3vlavn00iwe_oKns4f16TItXUlsbbzxpt0cQlrhM ref. ^64^ and https://datadryad.org/stash/share/HMwv5E4ZfHAieT3X5K5l6mLq-LogVb-HnTQF3VinSWo ref. ^65^, respectively. Source data are provided with this paper.
