# Author Correction: ASS1 metabolically contributes to the nuclear and cytosolic p53-mediated DNA damage response

**DOI:** 10.1038/s42255-024-01090-z

**Published:** 2024-07-04

**Authors:** Lisha Qiu Jin Lim, Lital Adler, Emma Hajaj, Leandro R. Soria, Rotem Ben-Tov Perry, Naama Darzi, Ruchama Brody, Noa Furth, Michal Lichtenstein, Elizabeta Bab-Dinitz, Ziv Porat, Tevie Melman, Alexander Brandis, Sergey Malitsky, Maxim Itkin, Yael Aylon, Shifra Ben-Dor, Irit Orr, Amir Pri-Or, Rony Seger, Yoav Shaul, Eytan Ruppin, Moshe Oren, Minervo Perez, Jordan Meier, Nicola Brunetti-Pierri, Efrat Shema, Igor Ulitsky, Ayelet Erez

**Affiliations:** 1 Department of Molecular Cell Biology, Weizmann Institute of Science, Rehovot, Israel; 2 Department of Medicine D, Beilinson Hospital, Petah Tikva, Israel; 3 Telethon Institute of Genetics and Medicine, Pozzuoli, Italy; 4 Department of Immunology and Regenerative Biology, Weizmann Institute of Science, Rehovot, Israel; 5 Department of Molecular Neuroscience, Weizmann Institute of Science, Rehovot, Israel; 6 Department of Biochemistry and Molecular Biology, The Institute for Medical Research Israel-Canada, Faculty of Medicine, Hebrew University of Jerusalem, Jerusalem, Israel; 7 Department of Life Sciences Core Facilities, Weizmann Institute of Science, Rehovot, Israel; 8 The De Botton Protein Profiling Institute of the Nancy and Stephen Grand Israel National Center for Personalized Medicine, Weizmann Institute of Science, Rehovot, Israel; 9 Cancer Data Science Lab, Center for Cancer Research, National Cancer Institute, National Institute of Health, Bethesda, MD USA; 10 Department of Translational Medicine, Medical Genetics, University of Naples Federico II, Naples, Italy; 11 Scuola Superiore Meridionale (SSM, School of Advanced Studies), Genomics and Experimental Medicine Program, University of Naples Federico II, Naples, Italy

**Keywords:** Cancer metabolism, Medical genetics, Metabolism, DNA, Cell biology

Correction to: *Nature Metabolism* 10.1038/s42255-024-01060-5, published online 10 June 2024

In the version of the article initially published, Sergey Malitsky and Maxim Itkin were listed as the last authors. The author list has now been corrected and Sergey Malitsky and Maxim Itkin are the fourteenth and fifteenth authors in the HTML and PDF versions of the article.

