# Supplementary material for: ASS1 metabolically contributes to the nuclear and cytosolic p53-mediated DNA damage response
Source: Nat Metab. 2024 Jun 10;6(7):1294–309. doi: 10.1038/s42255-024-01060-5 (PMC11272581; doi:10.1038/s42255-024-01060-5)
Supplement: Supplementary file 1 — Reporting Summary [file 42255_2024_1060_MOESM1_ESM.pdf]

nature portfolio

Corresponding author(s): Ayelet Erez
Last updated by author(s): Apr 24, 2024

Reporting Summary

Nature Portfolio wishes to improve the reproducibility of the work that we publish. This form provides structure for consistency and transparency in reporting. For further information on Nature Portfolio policies, see our Editorial Policies and the Editorial Policy Checklist.

Statistics

For all statistical analyses, confirm that the following items are present in the figure legend, table legend, main text, or Methods section.

n/a	Confirmed	
☐	☒	The exact sample size (*n*) for each experimental group/condition, given as a discrete number and unit of measurement
☐	☒	A statement on whether measurements were taken from distinct samples or whether the same sample was measured repeatedly
☐	☒	The statistical test(s) used AND whether they are one- or two-sided<br>*Only common tests should be described solely by name; describe more complex techniques in the Methods section.*
☐	☒	A description of all covariates tested
☐	☒	A description of any assumptions or corrections, such as tests of normality and adjustment for multiple comparisons
☐	☒	A full description of the statistical parameters including central tendency (e.g. means) or other basic estimates (e.g. regression coefficient) AND variation (e.g. standard deviation) or associated estimates of uncertainty (e.g. confidence intervals)
☐	☒	For null hypothesis testing, the test statistic (e.g. *F*, *t*, *r*) with confidence intervals, effect sizes, degrees of freedom and *P* value noted<br>*Give P values as exact values whenever suitable.*
☒	☐	For Bayesian analysis, information on the choice of priors and Markov chain Monte Carlo settings
☒	☐	For hierarchical and complex designs, identification of the appropriate level for tests and full reporting of outcomes
☒	☐	Estimates of effect sizes (e.g. Cohen's *d*, Pearson's *r*), indicating how they were calculated

Our web collection on statistics for biologists contains articles on many of the points above.

Software and code

Policy information about availability of computer code

Data collection	ImageJ v1.54i<br>CytoFLEX flow cytometer<br>MaxQuant v1.6.6.0<br>ImageLab 6.1<br>Zen 3.7<br>ChemiDoc MP Imaging System<br>GelDoc XR+ System<br>Novaseq platform<br>StepOnePlus Real-Time PCR Systems
Data analysis	Data analysis is described in the Methods<br>ImageJ v1.54i<br>ImageLab 6.1<br>FlowJo v10.10<br>R version 4.2.3<br>DESseq2<br>bowtie2

For manuscripts utilizing custom algorithms or software that are central to the research but not yet described in published literature, software must be made available to editors and reviewers. We strongly encourage code deposition in a community repository (e.g. GitHub). See the Nature Portfolio guidelines for submitting code & software for further information.

Data

Policy information about availability of data

All manuscripts must include a data availability statement. This statement should provide the following information, where applicable:

- Accession codes, unique identifiers, or web links for publicly available datasets
- A description of any restrictions on data availability
- For clinical datasets or third party data, please ensure that the statement adheres to our policy

RNA Seq: https://datadryad.org/stash/share/_SN3vlavn00iwe_oKns4f16TItXUlsbbzxpt0cQlrhM
Proteomics: https://datadryad.org/stash/share/HMwv5E4ZfHAieT3X5K5l6mLq-LogVb-HnTQF3VinSWo
Human genome hg38

Research involving human participants, their data, or biological material

Policy information about studies with human participants or human data. See also policy information about sex, gender (identity/presentation), and sexual orientation and race, ethnicity and racism.

Reporting on sex and gender	N/A
Reporting on race, ethnicity, or other socially relevant groupings	N/A
Population characteristics	N/A
Recruitment	N/A
Ethics oversight	No human subjects were involved in the study.

Note that full information on the approval of the study protocol must also be provided in the manuscript.

Field-specific reporting

Please select the one below that is the best fit for your research. If you are not sure, read the appropriate sections before making your selection.

☒ Life sciences ☐ Behavioural & social sciences ☐ Ecological, evolutionary & environmental sciences

For a reference copy of the document with all sections, see nature.com/documents/nr-reporting-summary-flat.pdf

Life sciences study design

All studies must disclose on these points even when the disclosure is negative.

Sample size	No statistical methods were used to pre-determine sample sizes but the sample size was chosen in advance based on common practice of the described experiment in the literature and is specified for each experiment. For cell culture experiments, at least 3 replicates per group and for animal studies at least 5 animals per group were used unless specified otherwise, taking into account the variability within a cage and experimental group as well as between individual experimental repetitions to set appropriate sample numbers to allow for sound interpretation of experimental results.
Data exclusions	No data was excluded from the analyses
Replication	Each experiment was conducted with biological and technical replicates and repeated at least three times for cell cultures studies unless specified otherwise. Trends were similar in all the replicates. All attempts at replication were successful.
Randomization	In cell culture experiments, cell dishes were randomly assigned to different study groups. In animal studies, mice were picked based on genotype and age-matched for the different study groups.
Blinding	Blinding was not carried out in cell culture experiments or in data collection, in which all experiments had to be carried out by the same researcher. Mice were age-, gender- and genotype-matched. For in vitro experiments, blinding was not relevant.

Reporting for specific materials, systems and methods

We require information from authors about some types of materials, experimental systems and methods used in many studies. Here, indicate whether each material, system or method listed is relevant to your study. If you are not sure if a list item applies to your research, read the appropriate section before selecting a response.

Materials & experimental systems

n/a	Involved in the study	
☐	☒	Antibodies
☐	☒	Eukaryotic cell lines
☒	☐	Palaeontology and archaeology
☐	☒	Animals and other organisms
☒	☐	Clinical data
☒	☐	Dual use research of concern
☒	☐	Plants

Methods

n/a	Involved in the study	
☒	☐	ChIP-seq
☐	☒	Flow cytometry
☒	☐	MRI-based neuroimaging

Antibodies

Antibodies used

Phospho-Histone H2A.X (Ser139) Antibody #2577 CST CST-2577S
Anti-ASS1 antibody [2B10] Abcam ab124465
Recombinant Anti-ASS1 antibody [EPR12398] Abcam ab170952
Anti-SMARCC1/BAF155 antibody Abcam ab126180
SMARCB1/BAF47 (D8M1X) Rabbit mAb #91735 CST CST-91735S
p53 (DO-1) Santa Cruz Biotechnology SC-126
Recombinant Anti-p21 antibody [EPR362] Abcam ab109520
Anti-GAPDH antibody [6C5] Abcam ab8245
Monoclonal Anti-α-Tubulin antibody produced in mouse Sigma Aldrich T5168-100UL, clone B-5-1-2
Go-ChIP-Grade Purified anti-Histone H3 (C-terminus) Biolegend BLG-819414
Anti-Importin 7 antibody Abcam ab99273
PerCP/Cyanine5.5 anti-H2A.X-Phosphorylated (Ser139) Antibody Biolegend BLG_613414
Anti-Lamin B1 antibody - Nuclear Envelope Marker Abcam ab16048
Recombinant Anti-MEK1 + MEK2 antibody [EPR16667] Abcam ab178876
Anti-2SC antibody Cambridge Research Biochemicals crb2005017d/6773
Alexa Fluor® 488 AffiniPure F(ab')$_2$ Fragment Donkey Anti-Rabbit IgG (H+L) Jackson ImmunoResearch Company 711-546-152
Recombinant Rabbit IgG Abcam ab172730
Alexa Fluor 594 Abcam ab150080
CPS1 Abcam ab45956
Vinculin Cell Signaling Technology CST-13901S
Donkey Anti-Rabbit IgG H&L (HRP) preadsorbed Abcam (ab97085)
Donkey Anti-Mouse IgG H&L (HRP) preadsorbed Abcam (ab98799)

Validation

All antibodies were purchased and validated by the supplier

Phospho-Histone H2A.X (Ser139) Antibody #2577 CST CST-2577S: Phospho-H2A.X (Ser139) Antibody detects endogenous levels of H2A.X only when phosphorylated at Ser139.
PMID: 20493860, 9488723, 19092802, 19234442

Anti-ASS1 antibody [2B10] Abcam ab124465. Positive Control Human ASS1 recombinant protein; HAP1, A431, Raji, L1210, MOLT4, Jurkat, A549, NIH 3T3, PC12 and COS7 cell lysates.
PMID: 35406678, 36539415, 33369168

Recombinant Anti-ASS1 antibody [EPR12398] Abcam ab170952 Positive Control: HAP1, HeLa, HepG2 cell lysates. Human fetal kidney and liver tissue lysates. Mouse liver and kidney lysates. Rat liver lysate. PMID: 33979616, 30429607, 26560030

Anti-SMARCC1/BAF155 antibody Abcam ab126180 Positive Control WB: HeLa, Neuro-2a, C8-D30, NIH/3T3, RAW 264.7, C2C12, HepG2, A431, HEK-293T lysate
PMID: 34249931,

SMARCB1/BAF47 (D8M1X) Rabbit mAb #91735 CST CST-91735S SMARCB1/BAF47 (D8M1X) Rabbit mAb recognizes endogenous levels of total SMARCB1/BAF47 protein.
PMID: 20110991, 9671307, 18508602

p53 (DO-1) Santa Cruz Biotechnology SC-126. Positive Controls: A549 cell lysate, Daudi cell lysate, NTERA-2 cl.D1 whole cell lysate
PMID: 10612830, 10618703, 36973430, 33397955

Recombinant Anti-p21 antibody [EPR362] Abcam ab109520 Positive Control WB: MCF7, HeLa, HEK293, HUVEC, LnCaP, U87 MG or HEK-293T cell lysates
PMID: 36305972, 34773073, 34773075

Anti-GAPDH antibody [6C5] Abcam ab8245 Positive Control WB: HeLa, A431, Jurkat, HEK-293, Raji whole cell lysate.
PMID: 35230639, 36121569, 36214976,

Monoclonal Anti-α-Tubulin antibody produced in mouse Sigma Aldrich T5168-100UL, clone B-5-1-2
PMID: 27447976, 31523176, 26735018

Go-ChIP-Grade Purified anti-Histone H3 (C-terminus) Biolegend BLG-819414 Each lot of this antibody is quality control tested by intracellular immunofluorescent staining with flow cytometric analysis

Anti-Importin 7 antibody Abcam ab99273 Positive Control: HeLa whole cell lysate, 293T whole cell lysate and Mouse NIH3T3 whole cell lysate.
PMID: 35196484, 35588577, 33869982

PerCP/Cyanine5.5 anti-H2A.X-Phosphorylated (Ser139) Antibody Biolegend BLG_613414: ach lot of this antibody is quality control tested by intracellular immunofluorescent staining with flow cytometric analysis.
PMID: 23449797, 18599611, 18522989

Anti-Lamin B1 antibody - Nuclear Envelope Marker Abcam ab16048 Positive Control: HeLa, PC12 and NIH/3T3 whole cell lysate. Wild type HAP1 whole cell lysate. Wild type HAP1 nuclear lysate. Human Pancreatic cell line whole cell lysate.
PMID: 36048349, 36436562, 36439629

Recombinant Anti-MEK1 + MEK2 antibody [EPR16667] Abcam ab178876 Positive Control: Jurkat, Daudi, HeLa, 293T, A549 and A431 whole cell lysates; Human fetal brain, heart, kidney and spleen lysates; Mouse and Rat brain and heart lysates
PMID: 35186499, 35428350, 35543207

Anti-2SC antibody Cambridge Research Biochemicals crb2005017d/6773
PMID: 16624247, 17726021

Alexa Fluor® 488 AffiniPure F(ab')$_2$ Fragment Donkey Anti-Rabbit IgG (H+L) Jackson ImmunoResearch Company 711-546-152
PMID: 37973910, 36970754, 37587337

Recombinant Rabbit IgG Abcam ab172730
PMID: 36116506, 36473367, 36478958

Goat Anti-Rabbit IgG H&L (Alexa Fluor® 594) Abcam ab150080
PMID: 37014018, 37051644, 37069582

CPS1 Abcam ab45956
PMID: 35655758, 33369168, 34059723

Vinculin Cell Signaling Technology CST-13901S
PMID: 38041178, 38022577, 38020236

Donkey Anti-Rabbit IgG H&L (HRP) preadsorbed Abcam (ab97085)
PMID: 37352136, 35379807, 35628417

Donkey Anti-Mouse IgG H&L (HRP) preadsorbed Abcam (ab98799)
PMID: 37352136, 35379807, 35883451

Eukaryotic cell lines

Policy information about cell lines and Sex and Gender in Research

Cell line source(s)	HCT116 WT - Moshe Oren (WIS)<br>HCT116 p53 EV - Moshe Oren (WIS)<br>HCT116 p53 KO - Moshe Oren (WIS)<br>HCT116 ASS1-KO - Generated in the lab<br>CTLN1 Fibroblasts - Coriell Institute for Medical Research GM08108<br>Normal Fibroblasts - Nicola Brunetti-Pierri/ATCC PCS-201-012<br>LS-174-T - Deborah Fass (WIS)<br>SKOV3 - ATCC Cat#HTB-77, RRID:CVCL_0532<br>MC38 - Moshe Oren (WIS)
Authentication	All the cell lines used showed similar feature to those reported in the literature. HCT116 ASS1-KO was validated using RTPCR, protein expression and enzymatic assay (XTT with or without arginine/citrulline). HCT116 p53-KO was validated using protein expression of p53. CTLN1 and normal fibroblasts were authenticated by testing protein expression of ASS1. MC38 and LS-174-T were also validated by protein expression of p53
Mycoplasma contamination	All cells were tested routinely for Mycoplasma using Mycoplasma EZ-PCR test kit (#20-700-20, Biological Industries, Kibbutz Beit Ha'emek). All cell lines were were tested negative to mycoplasma.
Commonly misidentified lines<br>(See ICLAC register)	There were no commonly misidentified cell lines used in this study.

Animals and other research organisms

Policy information about studies involving animals; ARRIVE guidelines recommended for reporting animal research, and Sex and Gender in Research

Laboratory animals	Tissue-specific knock out of ASS1 in hepatocytes was generated by crossing ASS1f/f C57BL/6 mice with transgenic mice overexpressing albumin (Alb) Cre. Asl Neo/Neo (B6.129S7-Asltm1Brle/J) mice were purchased from JAX.<br>Mice care practices are under sterile conditions, with sterile supplies. Mice were housed in individually ventilated cages, given ad libitum access to water and standard mouse chow with 12-hr light/dark cycles at 20-24°C and 30–70% relative humidity. Mice chosen for experiments were 10 weeks old.
Wild animals	There were no wild animals used.
Reporting on sex	All mice used were male.
Field-collected samples	There were no field-collected samples used.
Ethics oversight	Animal experiments were approved by the Weizmann Institute Animal Care and Use Committee Following US National Institute of Health, European Commission and the Israeli guidelines (IACUC 00260123-2). The authorization number from the Italian Ministry of Health is 413/2021-PR. Project number: CE571.64.

Note that full information on the approval of the study protocol must also be provided in the manuscript.

Flow Cytometry

Plots

Confirm that:

☒ The axis labels state the marker and fluorochrome used (e.g. CD4-FITC).

☒ The axis scales are clearly visible. Include numbers along axes only for bottom left plot of group (a 'group' is an analysis of identical markers).

☒ All plots are contour plots with outliers or pseudocolor plots.

☒ A numerical value for number of cells or percentage (with statistics) is provided.

Methodology

Sample preparation	For cell cycle analysis, cells were fixed with Cyto-FastTM Fix/Perm buffer (Biolegend, 426803) and DAPI (Biolegend, 422801,1:500) was used to stain the nucleus. For γH2AX staining, cells were fixed with Cyto-FastTM Fix/Perm buffer (Biolegend, 426803) and stained with antibody (γH2AX (CST-9718S), 1:100) overnight. After washing, cells were incubated with the Alexa-Fluor 488 antibody (Jackson ImmunoResearch Ltd, 711-546-152, 1:500) for one hour. DAPI (Biolegend, 422801, 1:500) was used to stain the nucleus. After washing, cells were resuspended in 100 µl of PBS and were collected using ImageStream and the analysis was performed on the IDEAS software.<br>HCT-116 cells were plated at 60% confluency and grown for 24 hrs in full DMEM. Dox treatment was performed as previously mentoned in Plasmax medium without Arginine and Asparate. Fumarate rescue was performed by adding fresh media with 1 mM fumarate (Alfa Aesar) in DMSO (final concentration 0.4%) or equal concentration of DMSO after a 2 hr incubation with 1 µg/ml Dox . Following 48 hrs incubation, cells were trypsinized, washed with PBS and stained with 400 nM Apotracker green (Biolegend 427402) and DAPI (Biolegend 422801)to detect apoptotic and dead cells. Data was acquired on CytoFLEX flow cytometer (Beckman coulter) and analyzed with FlowJo software (Tree Star). The early and the late apoptitic gated cells were combined to calculate the precent of apoptotic cells per sample.
Instrument	ImageStreamX mark II (Amnis, Part of Luminex, Au. TX)<br>CytoFLEX flow cytometer (Beckman coulter)
Software	IDEAS 6.3 software (Amnis, Part of Luminex, Au. TX).<br>FlowJo software (Tree Star)
Cell population abundance	No cells sort was preformed
Gating strategy	Live cells were gated according their signal of the DAPI staining (Channel 7). Cell doublets were then gated according to the area vs. Aspect Ratio (the ratio between the minor axis and the major axis of a best-fit ellipse for the nuclear object) of the bright-field image. Focused cells were selected using the Gradient RMS feature (measures the sharpness quality of an image by detecting large changes of pixel values in the image, computed using the average gradient of a pixel normalized for variations in intensity levels).<br>For Flow cytometry single cells were gated to remove doublets from the analysis. The FITC labeled cells with and without DAPI were gated to determine the percentage of the late and early apoptotic population.

☒ Tick this box to confirm that a figure exemplifying the gating strategy is provided in the Supplementary Information.
