# Supplementary figures and images for: ASS1 metabolically contributes to the nuclear and cytosolic p53-mediated DNA damage response
Source: Nat Metab. 2024 Jun 10;6(7):1294–309. doi: 10.1038/s42255-024-01060-5 (PMC11272581; doi:10.1038/s42255-024-01060-5)

Supp Fig 1B

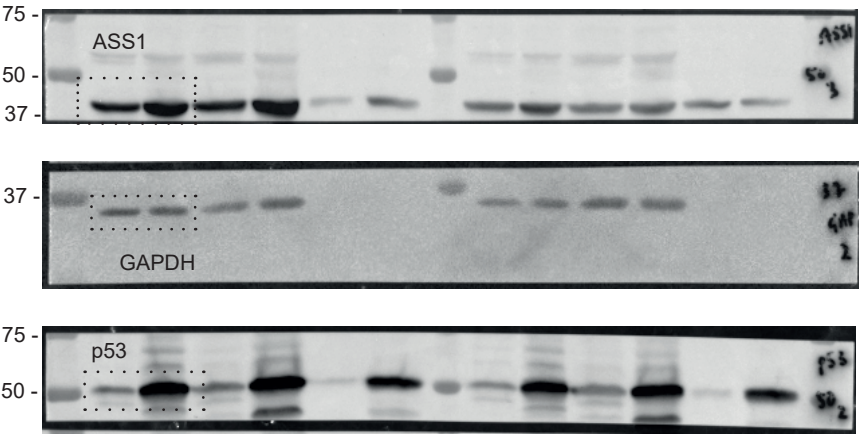

Supp Fig 1F

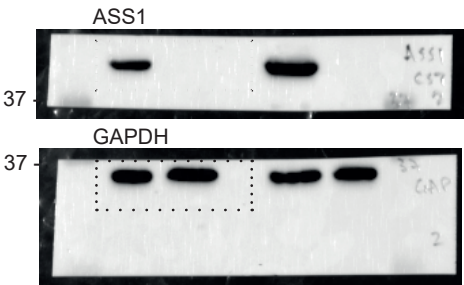

Supp Fig 2A

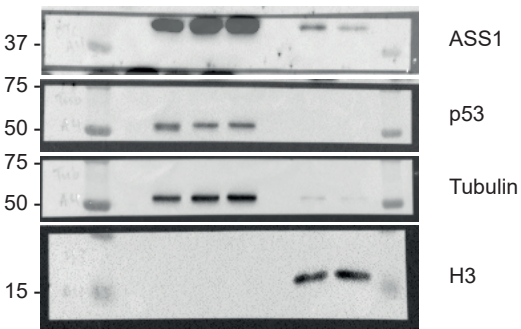

Supp Fig 2B

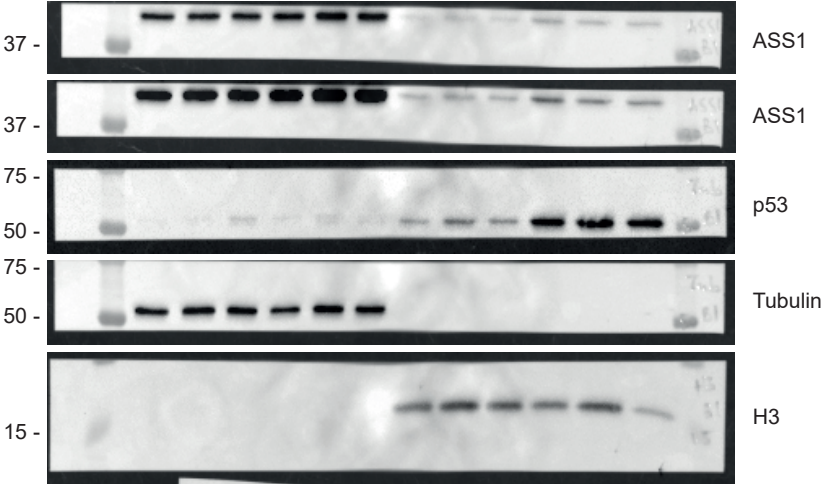

Supp Fig 2C

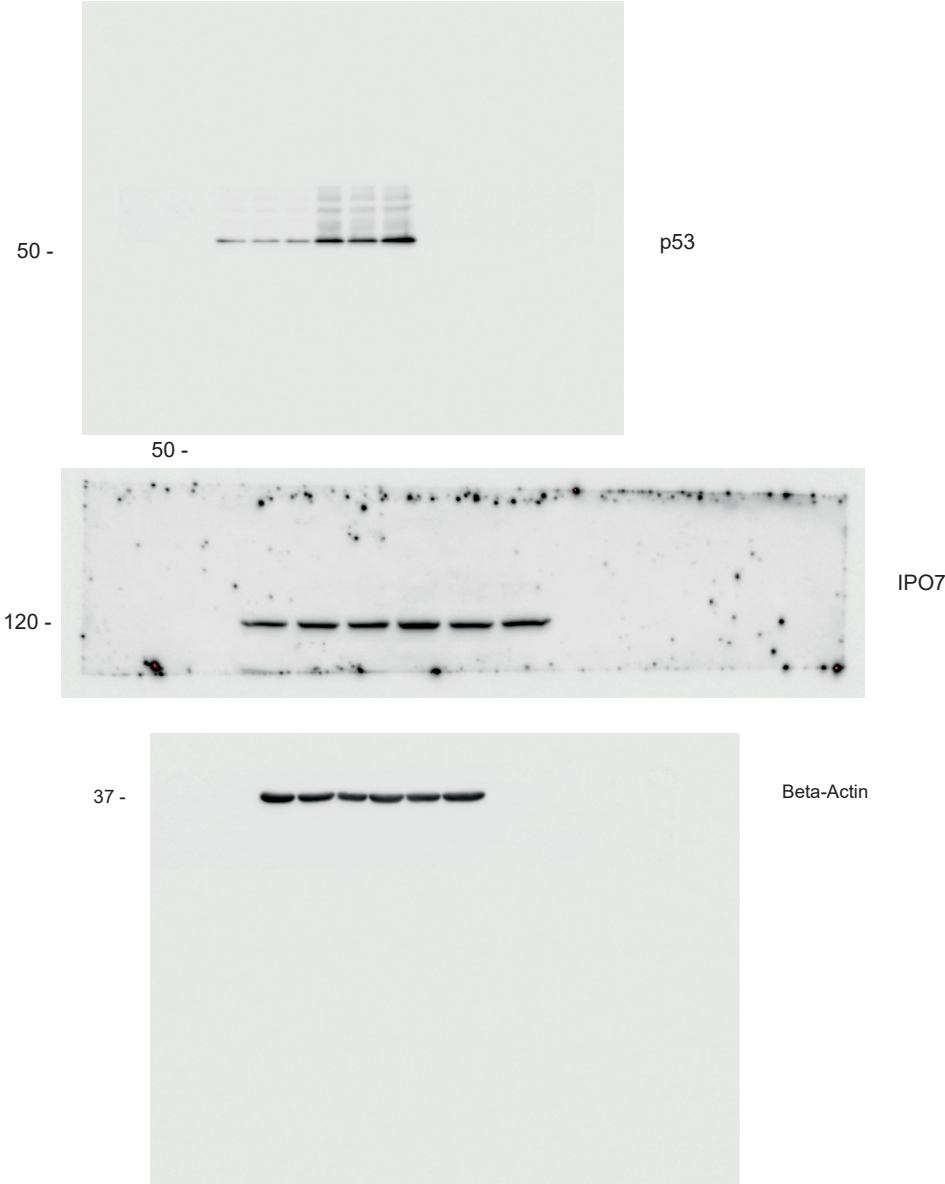

Supp Fig 2D

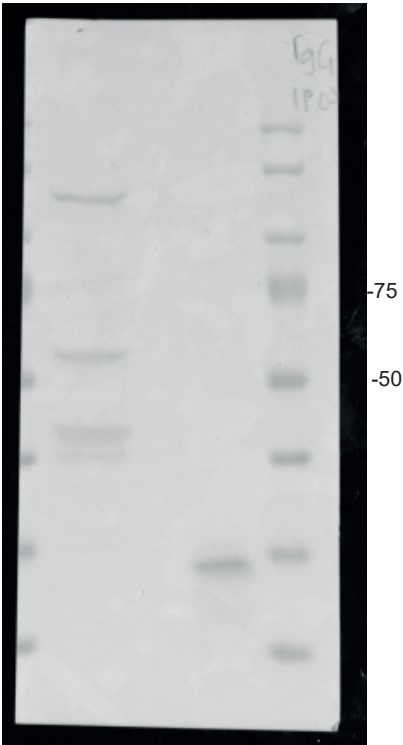

Supp Fig 3C

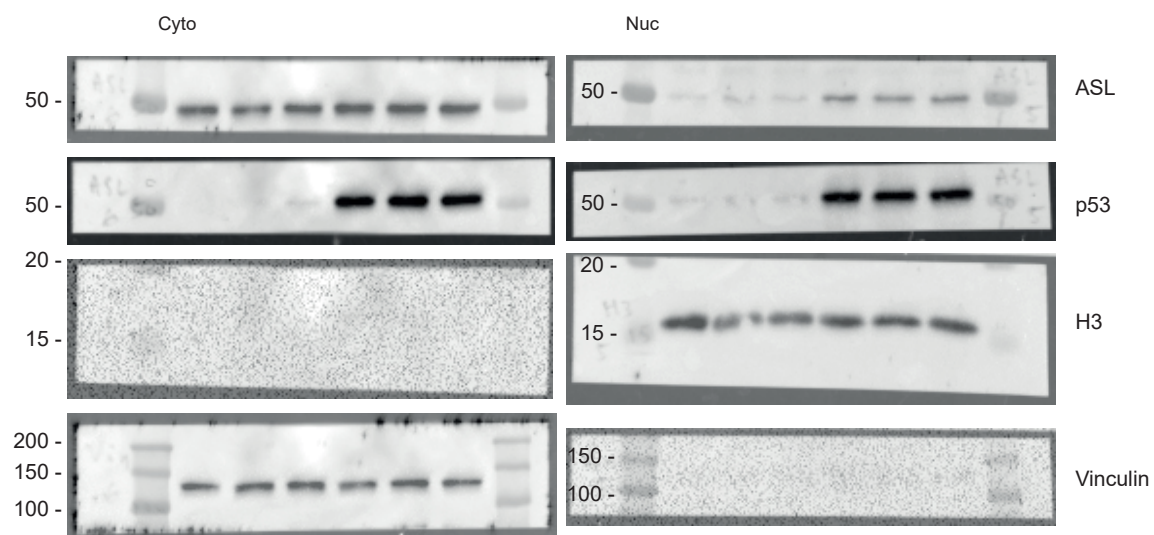

Supp. Fig. 4B

Ponceau Staining

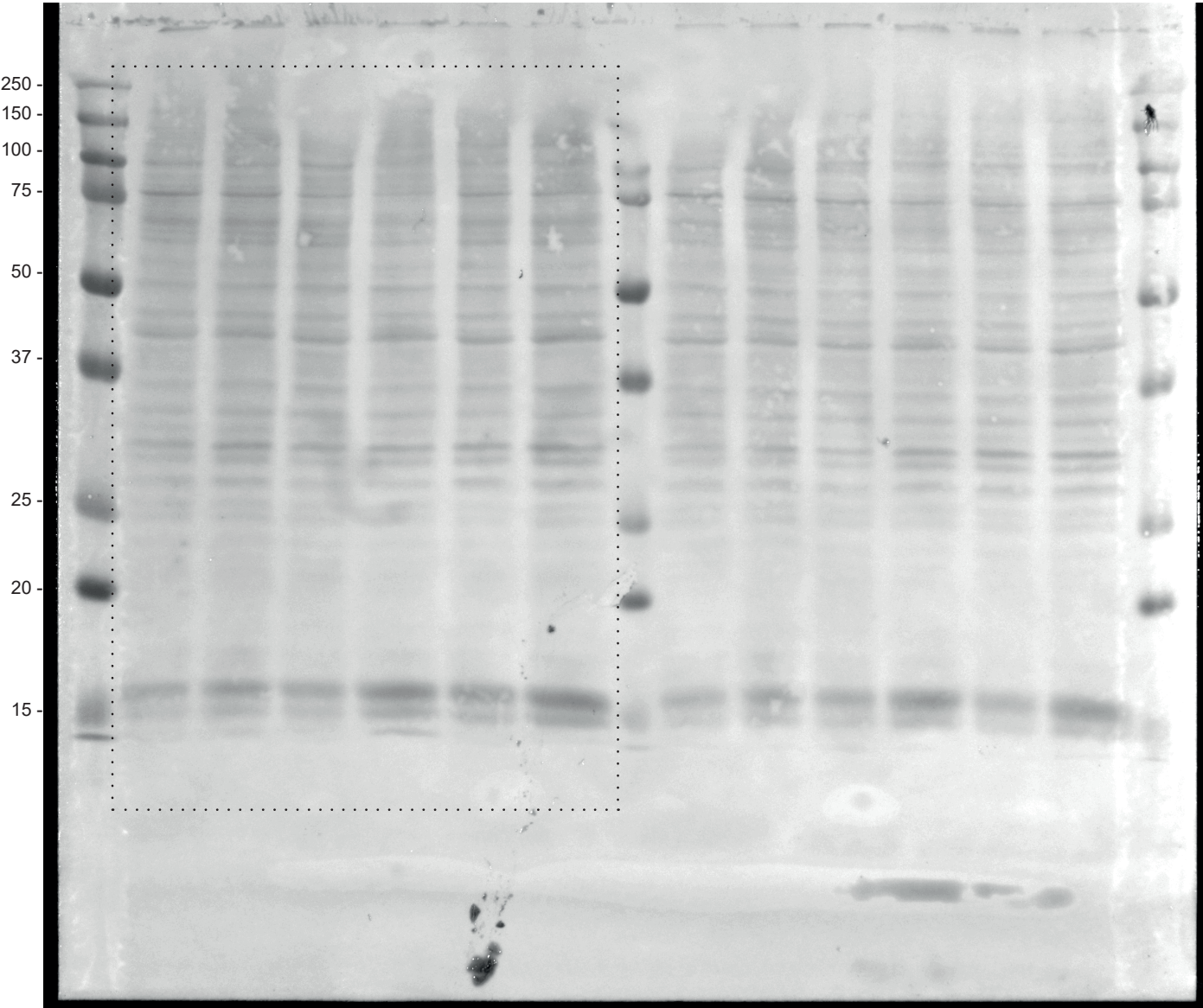

Supplement: Supplementary file 3 — Uncropped scans for Supplementary Figures. [file 42255_2024_1060_MOESM3_ESM.pdf]

Fig 1G

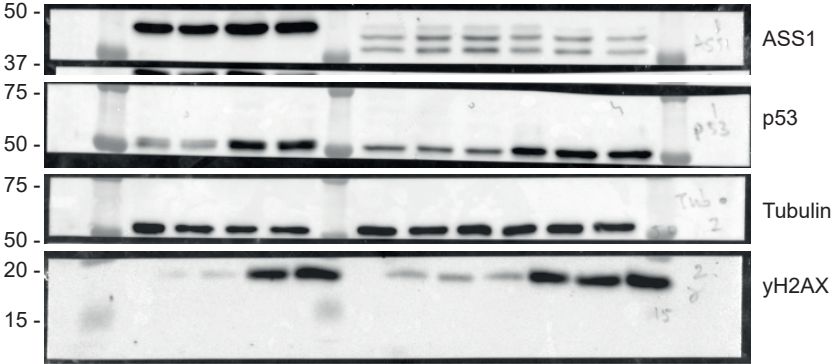

Supplement: Supplementary file 10 — Full-length, unprocessed gels or blots. [file 42255_2024_1060_MOESM10_ESM.pdf]

Fig 2B

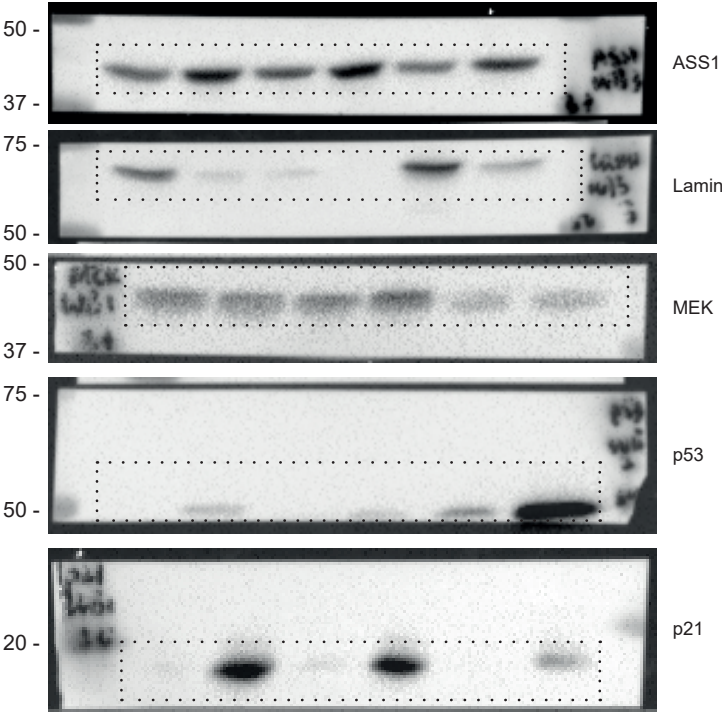

Fig 2C

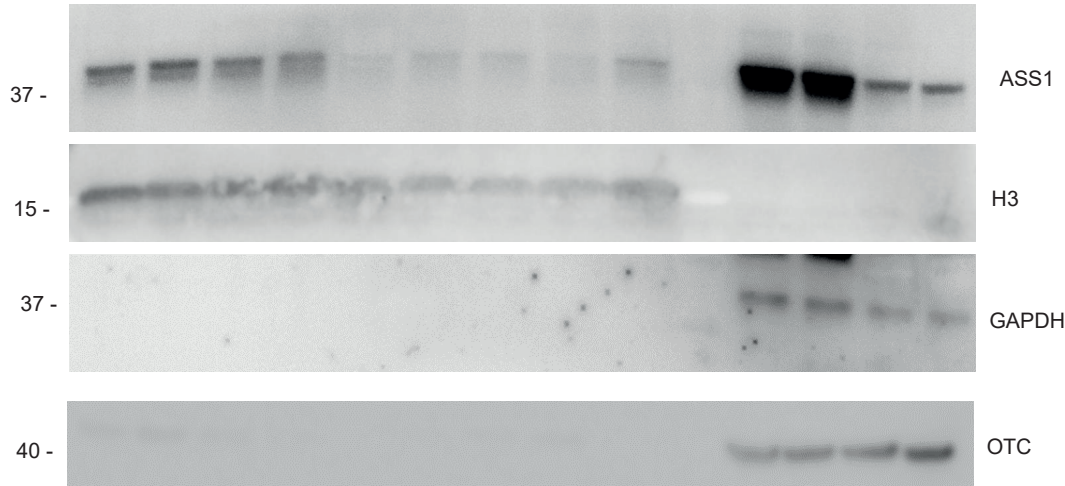

Fig 2D

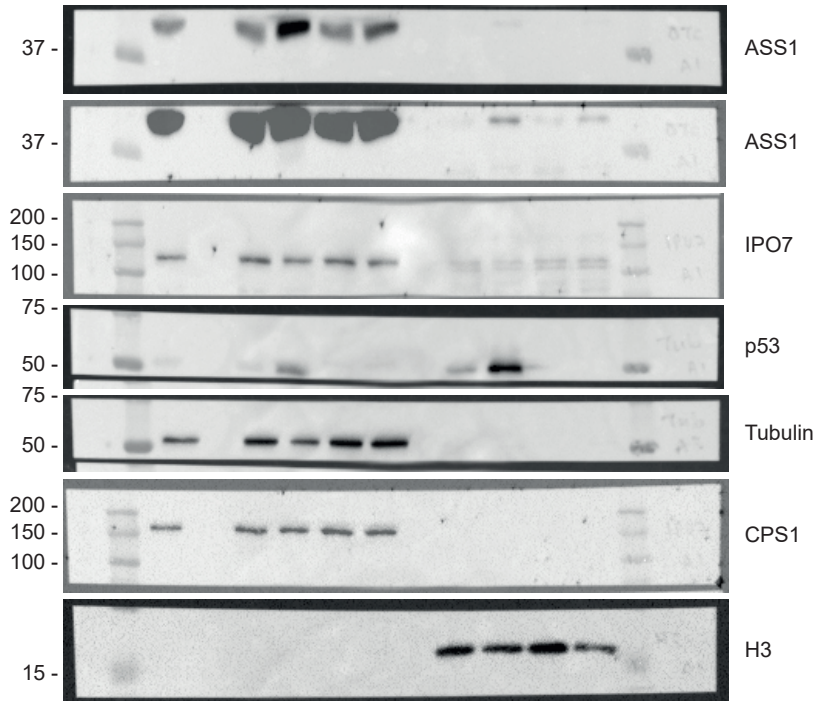

Fig 2F

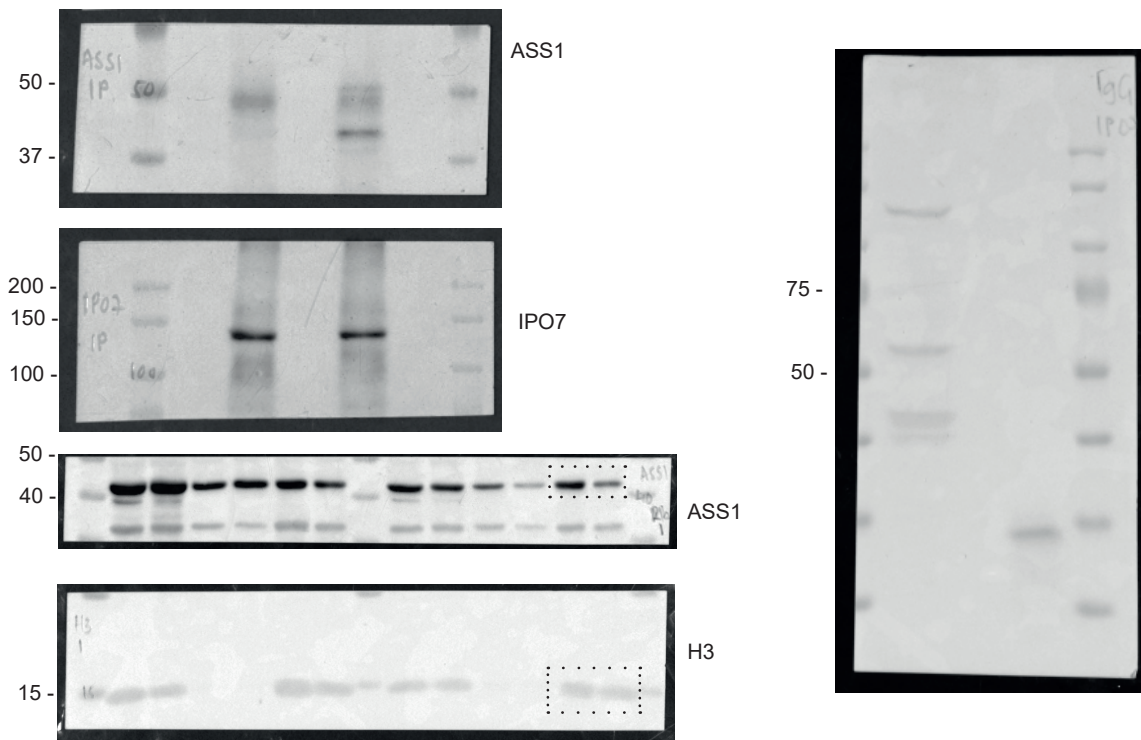

Fig 2G

Cytoplasmic

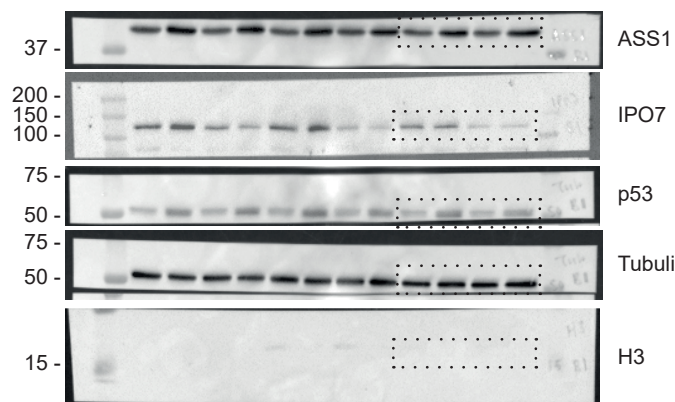

Nuclear

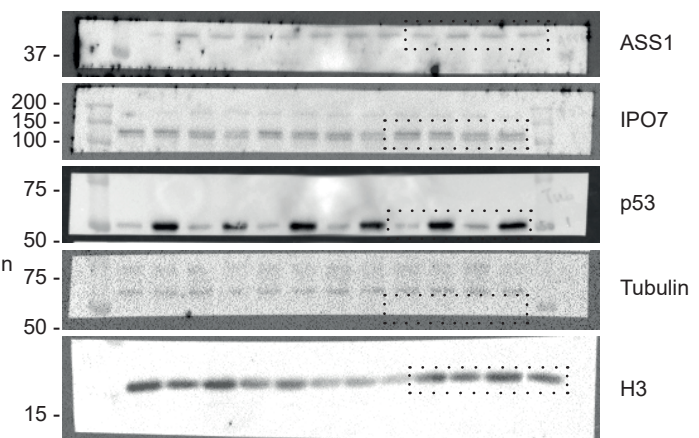

Supplement: Supplementary file 11 — Full-length, unprocessed gels or blots. [file 42255_2024_1060_MOESM11_ESM.pdf]

Fig 3D

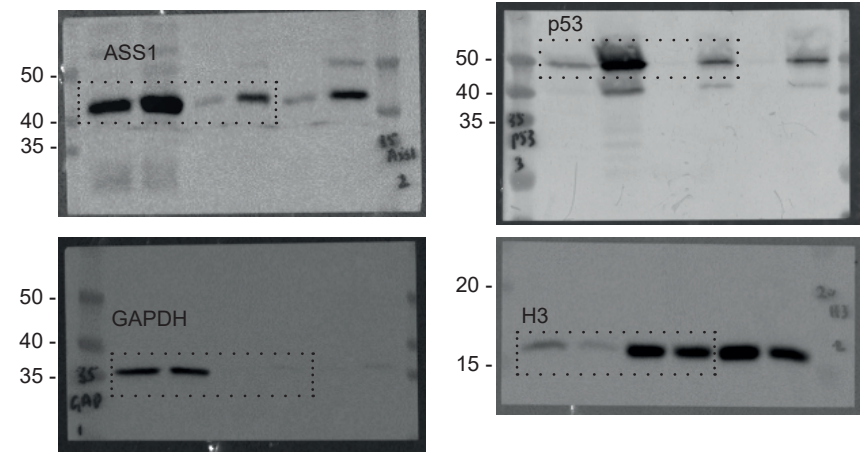

Fig 3E

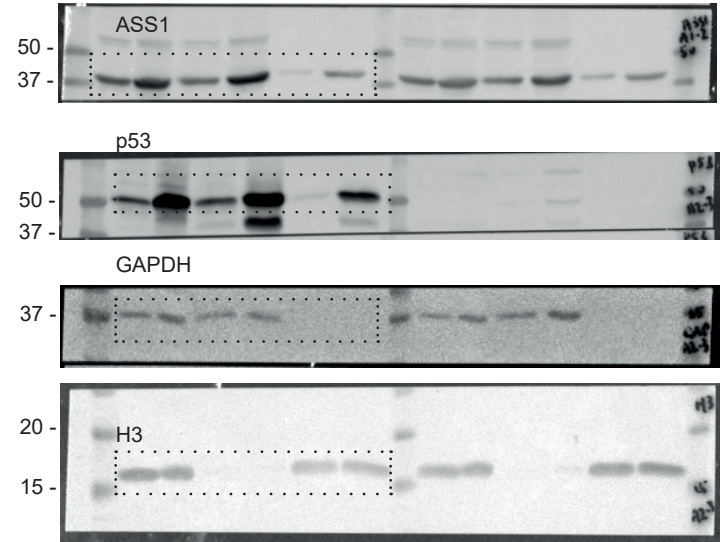

Supplement: Supplementary file 12 — Full-length, unprocessed gels or blots. [file 42255_2024_1060_MOESM12_ESM.pdf]

Fig 4A

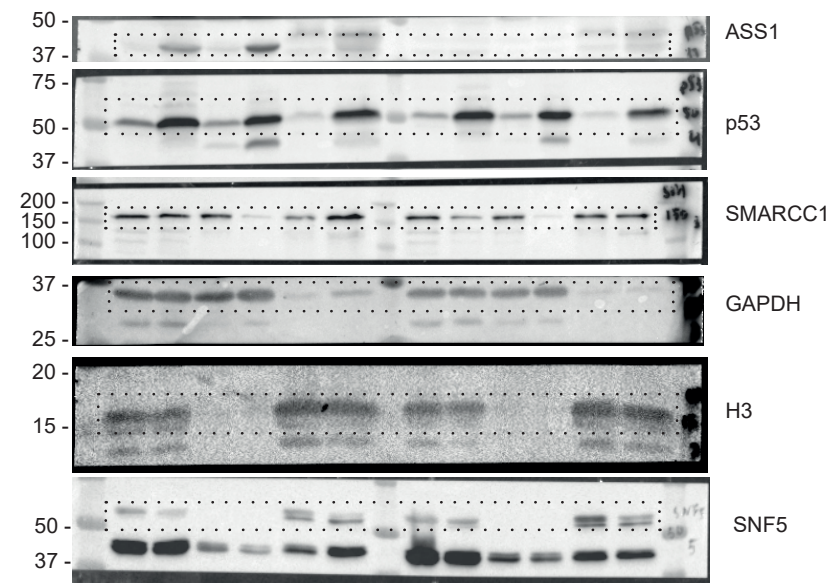

Fig 4B

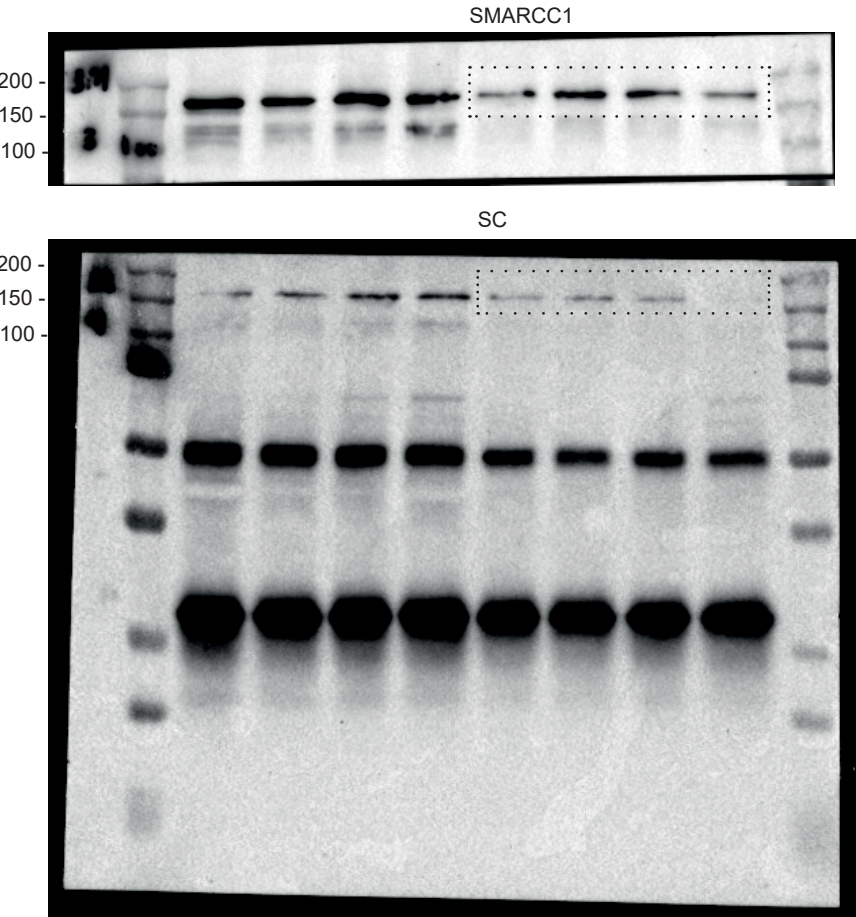

Fig 4C

Anti-2SC

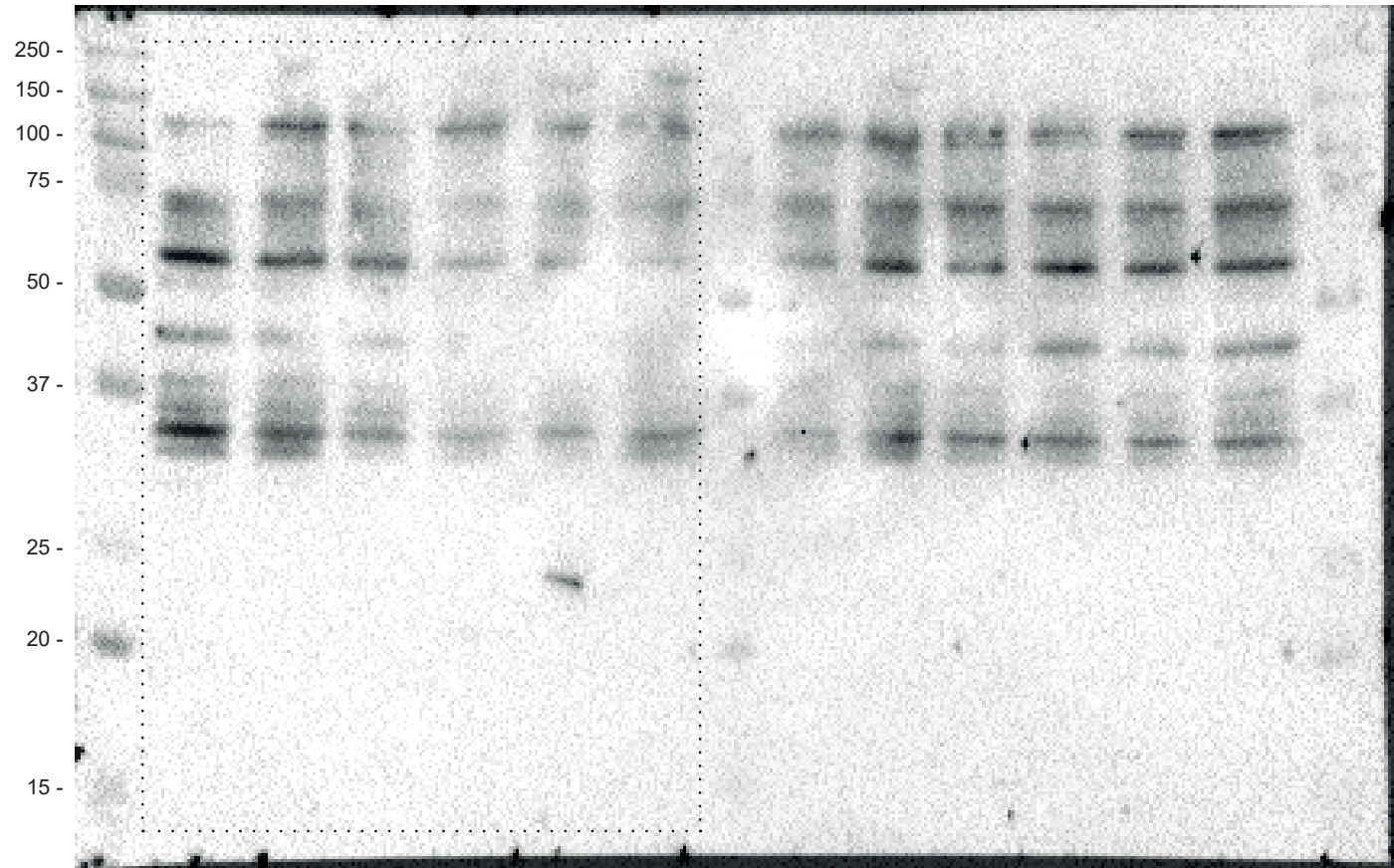

Fig 4D

SNF5

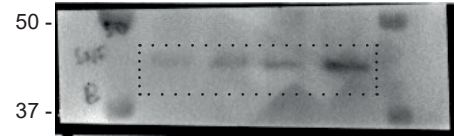

SMARCC1

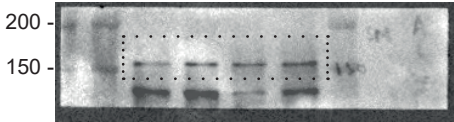

Supplement: Supplementary file 13 — Full-length, unprocessed gels or blots. [file 42255_2024_1060_MOESM13_ESM.pdf]
