# Supplementary material for: ASS1 metabolically contributes to the nuclear and cytosolic p53-mediated DNA damage response
Source: Nat Metab. 2024 Jun 10;6(7):1294–309. doi: 10.1038/s42255-024-01060-5 (PMC11272581; doi:10.1038/s42255-024-01060-5)

Gating strategy- **ImageStream**

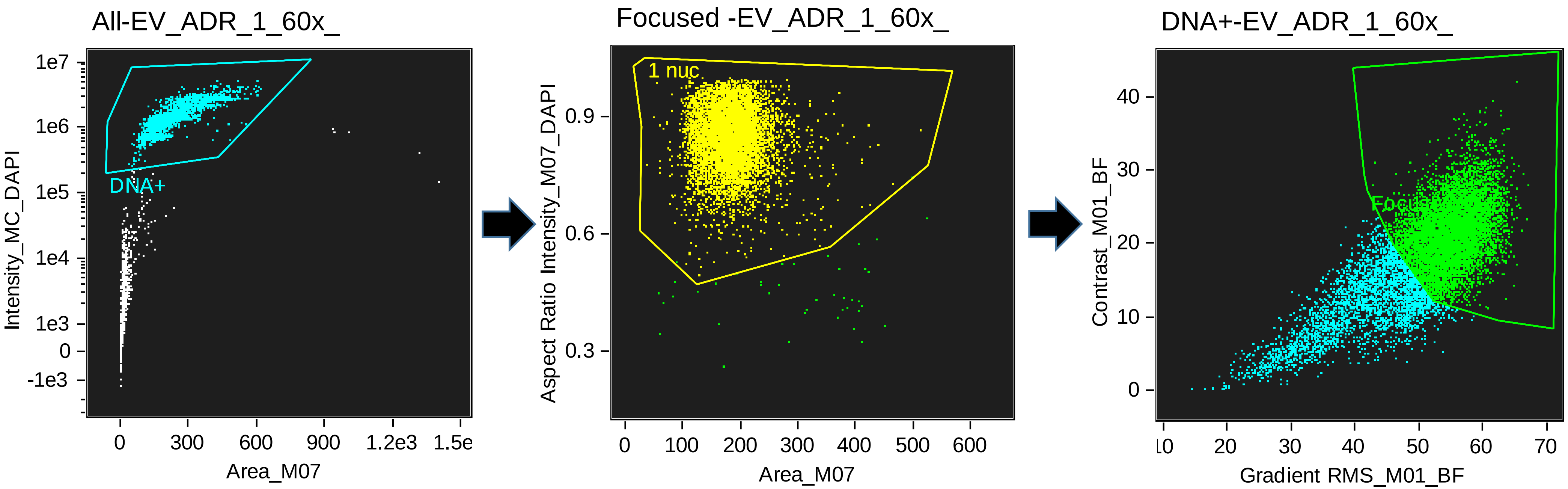

Area_M07, Intensity_MC_DAPI

Population	Count	%Gated
All	12173	100
DNA+	10275	84.4

Area_M07, Aspect Ratio Intensity_M07_DAPI

Population	Count	%Gated
Focused & DNA+	7256	100
1 nuc & Focused & DNA+	7214	99.4

Gradient RMS_M01_BF, Contrast_M01_BF

Population	Count	%Gated
DNA+	10275	100
Focused & DNA+	7256	70.6

Gating strategy flow cytometry -

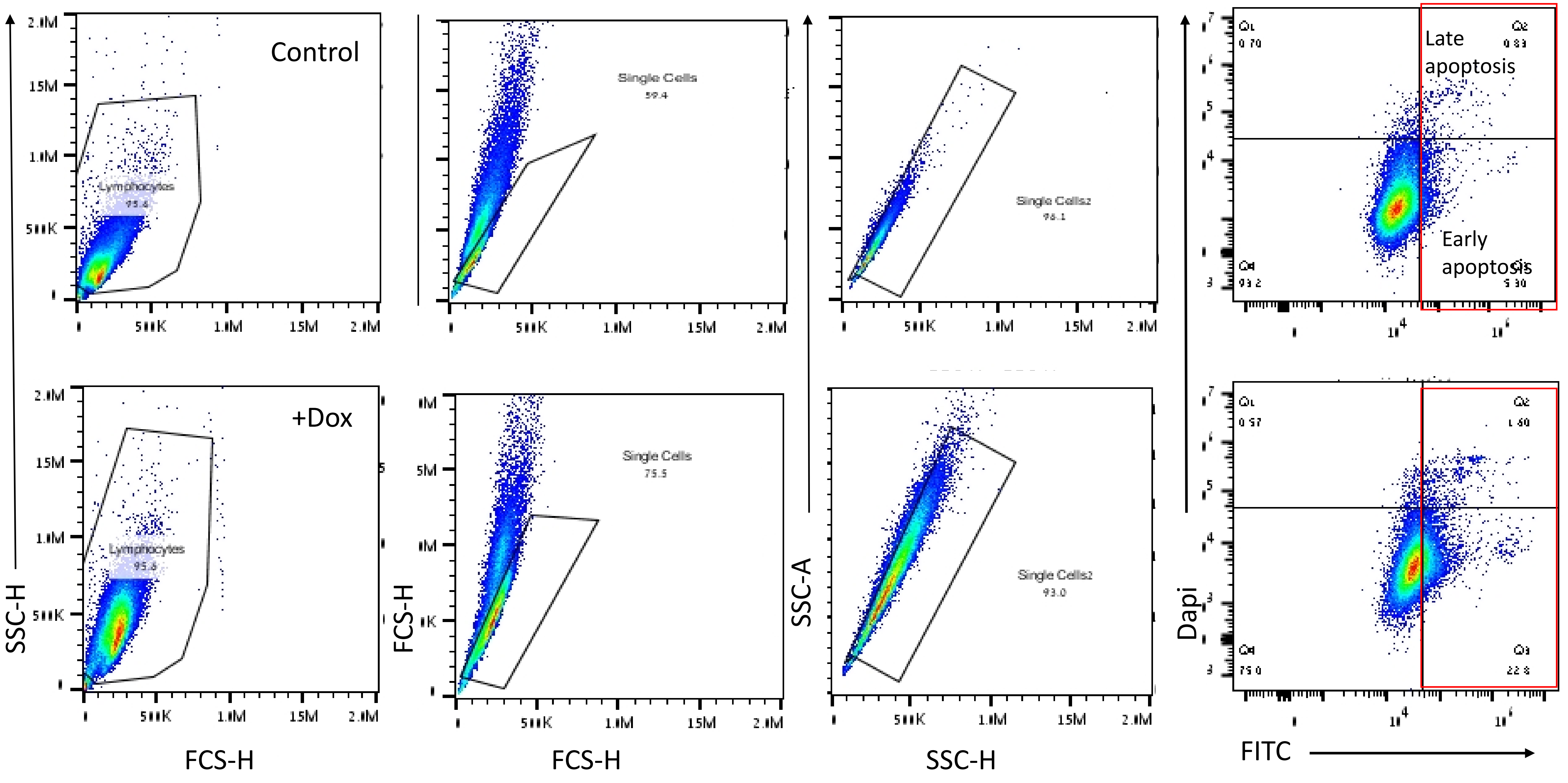

Supplement: Supplementary file 4 — Live cells were gated according to their signal of the DAPI staining (Channel 7). Cell doublets were then gated according to the area vs aspect ratio (the ratio between the minor axis and the major axis of a best-fit ellipse for the nuclear object) of the bright-field image. Focused cells were selected using the Gradient RMS feature (measures the sharpness quality of an image by detecting large changes of pixel values in the image, computed using the average gradient of a pixel normalized for variations in intensity levels). [file 42255_2024_1060_MOESM4_ESM.pdf]
